# Colon Transcriptomics Reveals Sex-Dependent Metabolic Signatures in Response to 2-Amino-1-methyl-6-phenylimidazo[4,5-b]pyridine Treatment in C57BL/6N Mice

**DOI:** 10.3390/ijms21186620

**Published:** 2020-09-10

**Authors:** Jeong Hoon Pan, Cara Cicalo, Brandy Le, Suwon Jeon, Sangyub Kim, Kyung A. Hwang, Byungwhi Kong, Jin Hyup Lee, Jae Kyeom Kim

**Affiliations:** 1 Department of Behavioral Health and Nutrition, College of Health Sciences, University of Delaware, Newark, DE 19716, USA; jhpan@udel.edu (J.H.P.); ccicalo@udel.edu (C.C.); brandyle@udel.edu (B.L.); swjeon@udel.edu (S.J.); 2 School of Human Environmental Sciences, Dale Bumpers College of Agricultural, Food and Life Sciences, University of Arkansas, Fayetteville, AR 72701, USA; 3 Department of Pharmacology, Penn State College of Medicine, Penn State University, Hershey, PA 17033, USA; sangyubkim5180@gmail.com; 4 Department of Agrofood Resources, National Institute of Agricultural Sciences, Rural Development Administration, Jeollabuk-do 55365, Korea; kah366@korea.kr; 5 Department of Poultry Science, Dale Bumpers College of Agricultural, Food and Life Sciences, University of Arkansas, Fayetteville, AR 72701, USA; bkong@uark.edu; 6 Department of Food and Biotechnology, College of Science & Technology, Korea University, Sejong 30019, Korea; jinhyuplee@korea.ac.kr

**Keywords:** colonic transcriptome, sex-based difference, lipolysis, mitochondrial dysfunction, PhIP metabolism

## Abstract

Diets high in red meats, particularly meats cooked at high temperature, increase the risk of colon cancer due to a production of heterocyclic aromatic amines (HAAs). Of the identified HAAs, 2-amino-1-methyl-6-phenylimidazo[4,5-b]pyridine (PhIP) is the most mass abundant colon carcinogen in charred meat or fish. Here, we comprehensively examined sex-dependent colon transcriptome signatures in response to PhIP treatment to identify biological discrepancies. Eight-week-old male and female C57BL/6N mice were intraperitoneally injected with PhIP (10 mg/kg of body weight) and colon tissues were harvested 24 h after PhIP injection, followed by colon transcriptomics analysis. A list of differentially expressed genes (DEGs) was utilized for computational bioinformatic analyses. Specifically, overrepresentation test using the Protein Analysis Through Evolutionary Relationships tool was carried out to annotate sex-dependent changes in transcriptome signatures after PhIP treatment. Additionally, the most significantly affected canonical pathways by PhIP treatment were predicted using the Ingenuity Pathway Analysis. As results, male and female mice presented different metabolic signatures in the colon transcriptome. In the male mice, oxidative phosphorylation in the mitochondrial respiratory chain was the pathway impacted the most; this might be due to a shortage of ATP for DNA repair. On the other hand, the female mice showed concurrent activation of lipolysis and adipogenesis. The present study provides the foundational information for future studies of PhIP effects on underlying sex-dependent mechanisms.

## 1. Introduction

Colorectal cancer is the third most common cancer worldwide, regardless of sex differences, and it is the 2nd most fatal cancer in the United States according to the most recent statistics data [1]. In 2020, approximately 150,000 new colorectal cancer diagnoses are expected in the United States, with more than 50,000 predicted deaths occurring in the same year [1]. Westernized diets have been linked to the incidence of colorectal cancer, particularly the consumption of diets rich in meats. Specifically, cooking meats at high temperatures generates heterocyclic aromatic amines (HAAs), potent carcinogens [2,3,4]. Of the identified HAAs, 2-amino-1-methyl-6-phenylimidazo[4,5-b]pyridine (PhIP) is the most abundant HAA produced in charred meat and fish. Hence, PhIP and its early cancer markers, such as colonic PhIP-DNA adducts and aberrant crypt foci, have been widely utilized in various animal models for colon cancer studies (e.g., Reference [5]).

PhIP is a procarcinogen, hence metabolic activation is required to induce colon cancer; activation of PhIP is mainly initiated through *N*-hydroxylation by cytochrome P450 (CYP) 1A2 generating *N*^2^-hydroxy-PhIP. The activated PhIP metabolite is further esterified by *N*-acetyltransferase 2 and sulfotransferase to *N*^2^-acetoxy-PhIP and *N*^2^-sulfonyloxy-PhIP, respectively [6,7,8]. These conjugated metabolites can produce electrophilic nitrenium ions which can form PhIP-DNA adducts. The formation of colonic PhIP-DNA adducts is the initial step of colon carcinogenesis [9]. On the other hand, *N*^2^-hydroxy-PhIP can be detoxified by glucuronidation and glutathionylation by uridine 5′-diphospho-glucuronosyltransferase and glutathione *S*-transferase, respectively, thereby catalyzing detoxification and efficient excretion of PhIP [8,10,11,12].

Despite extensive research on the molecular mechanisms of PhIP in the context of colonic carcinogenesis, there is a lack of information as to changes in colonic transcriptome signatures resulting from PhIP exposure. Unraveling global gene expression signatures may provide additional insight into underlying mechanisms modulated by PhIP treatment. Moreover, sex-dependent responses against PhIP in colon tissue need more attention since there are previous studies showing that males and females respond differently to PhIP in a rodent model [13,14,15]. For instance, chronic exposure to PhIP induced colon tumors in only male F344 rats [15]. Another study reported opposing findings that female mice are more susceptible to induced lymphomas in the hematopoietic system of CDF_1_ mice [14]. Chen et al. recently profiled microRNAs in response to PhIP treatment but the study (1) provided functional annotation via prediction of target transcripts and (2) female rats were not included to define the microRNA signature [16]. Therefore, in this study, we aimed (1) to characterize colonic transcriptome signatures in mice and (2) to compare sex-dependent responses in colon tissues resulting from PhIP treatment using computational bioinformatics tools.

## 2. Results

### 2.1. Identification of DEGs in Response to PhIP Injection

**Figure 1:**
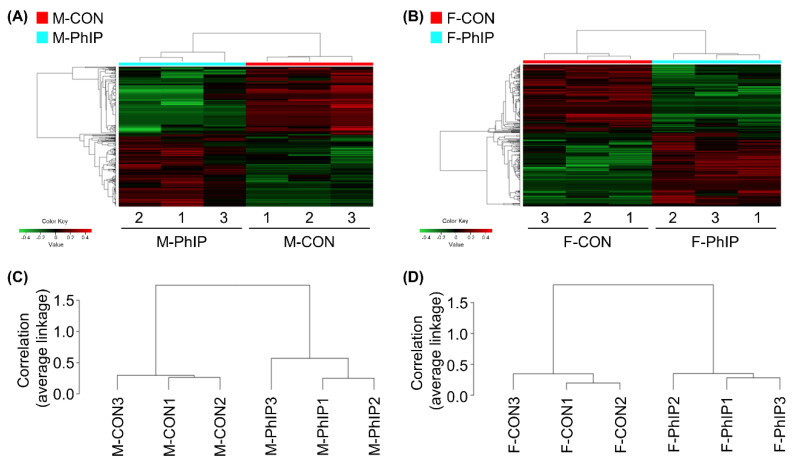
Hierarchical clustering and heatmap for differentially expressed genes (DEGs) of colon transcriptome profiles. (**A**,**B**) Heatmap and hierarchical clustering of log2 fold changes indicate DEGs (rows) between control and 2-amino-1-methyl-6-phenylimidazo[4,5-b]pyridine (PhIP) groups in (**A**) male and (**B**) female. Red keys indicate an increased gene expression in PhIP-treated group compared to the control, whereas green keys indicate down-regulation. (**C**,**D**) Dendrogram of hierarchical clustering, performed with the Pearson’s correlation coefficient and the average linkage method, which shows the interclass correlation between control and PhIP groups in (**C**) male and (**D**) female. M-PhIP, male mice treated with PhIP; M-CON, control male mice; F-PhIP, female mice treated with PhIP; F-CON, control female mice.

From the transcriptomics dataset, a total of 11,341 transcripts remained after excluding genes with low read counts and applying normalization. Lists of differentially expressed genes (DEGs) for male and female groups were separately obtained based on the following cut-off criteria—fold change ≥1.2 and *p*-value < 0.05. Accordingly, we were able to select 651 and 493 DEGs for male and female groups, respectively (Tables S1 and S2). In male mice, 344 genes were up-regulated and 307 genes were down-regulated by acute PhIP exposure. On the other hand, we identified 254 up-regulated and 239 down-regulated genes in female mice treated with PhIP. To visualize transcriptomic differences between control (CON) and PhIP-treated groups in each sex, heatmaps were generated showing log_2_ fold change values (Figure 1A,B). To determine whether these gene expression profiles were different between the groups, we analyzed RNA sequencing (RNAseq) data of CON and PhIP-treated groups in each sex by hierarchical clustering analysis using the Pearson’s correlation coefficient and the average linkage method (Figure 1C,D).

### 2.2. Comparison of Sex-Dependent Gene Ontology of DEGs Using the PANTHER

First, DEGs from a combined transcriptomics dataset of male and female mice were annotated by analyzing Gene Ontology (GO) to explore sex-independent impacts of PhIP treatment on global gene expression. Next, GO terms were annotated regardless of sex difference—Exogenous drug catabolic process (GO:0042738, 7.31 increased fold enrichment in reads), Xenobiotic metabolic process (GO:0006805, 5.17 increased fold enrichment in reads) in Biological Process and Carboxylic ester hydrolase activity (GO:0052689, 4.18 increased fold enrichment in reads) in Molecular Function. These indicate that PhIP significantly enriched xenobiotic metabolism pathways (Table S3).

To pinpoint and differentiate key sex-dependent GO terms for the colon transcriptome in response to PhIP treatment, each DEG was subjected to the Protein Annotation through Evolutionary Relationships (PANTHER) over-representation test. The top 10-fold enriched GO terms in male mice were listed in Table S4. Overall terms in Biological Process, Molecular Function and Cellular Component are highly relevant to the mitochondrial respiratory chain including Electron Transport Chain (GO:0022900), Cytochrome-c Oxidase Activity (GO: 0004129) and Respiratory Chain Complex I (GO:0045271). On the other hand, GO terms in female mice were not as enriched as the GO terms for male mice (Table S5). Negative Regulation of Establishment of Protein Localization (GO:1904950) and Lipase Activity (GO:0016298) were the most fold enriched terms for Biological Process and Molecular Function, respectively. However, there were no terms for Cellular Component. This comparison clearly differentiated sex-dependent effects of PhIP on Biological Processes.

### 2.3. Comparison of Sex-Dependent Canonical Pathways of DEGs Using the IPA

**Figure 2:**
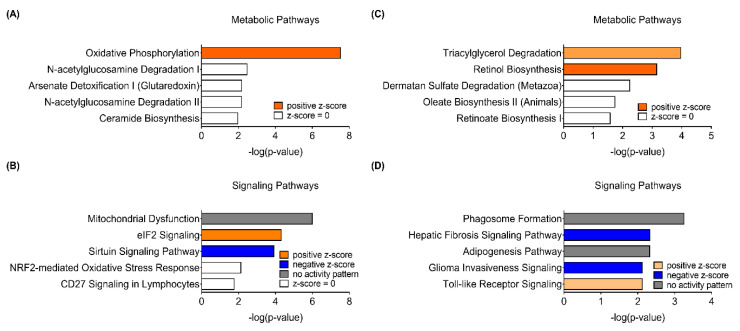
Canonical pathways retrieved from the colon transcriptome of male (**A**,**B**) and female (**C**,**D**) mice treated with 2-amino-1-methyl-6-phenylimidazo[4,5-b]pyridine (PhIP) using the Ingenuity Pathway Analysis (IPA) software. (**A**,**C**) Top five canonical metabolic pathways enriched in differentially expressed genes of colon transcriptome. (**B**,**D**) Top five canonical signaling pathways enriched in differentially expressed genes of the colon transcriptome.

In addition to the GO analysis, respective DEGs were utilized for Ingenuity Pathway Analysis (IPA) Core Analysis to compare how sex differently impacts canonical pathways in response to PhIP treatment. Of note, in male mice, Oxidative Phosphorylation (OXPHOS) and Mitochondrial Dysfunction were the top canonical metabolic pathway (Figure 2A) and canonical signaling pathway (Figure 2B), respectively, influenced the most by PhIP. On the other hand, in female mice, Triacylglycerol Degradation was the most significant canonical metabolic pathway followed by Retinol Biosynthesis, Dermatan Sulfate Degradation, Oleate Biosynthesis II and Retinoate Biosynthesis I (Figure 2C). Moreover, Phagosome Formation, Hepatic Fibrosis Signaling Pathway, Adipogenesis Pathway, Glioma Invasiveness Signaling and Toll-like Receptor Signaling were the top five canonical signaling pathways in female mice (Figure 2D). Genes involved in key canonical pathways are listed in Table 1. The IPA Canonical Pathway analysis shows different canonical pathways between male and female mice. Specifically, there was no change in lipid metabolism in male mice and none of the pathways in female mice were related to mitochondria. These IPA results are somewhat in line with the results of GO analysis.

### 2.4. Quantitative RT-PCR Validation for Predicted Canonical Pathways

**Figure 3:**
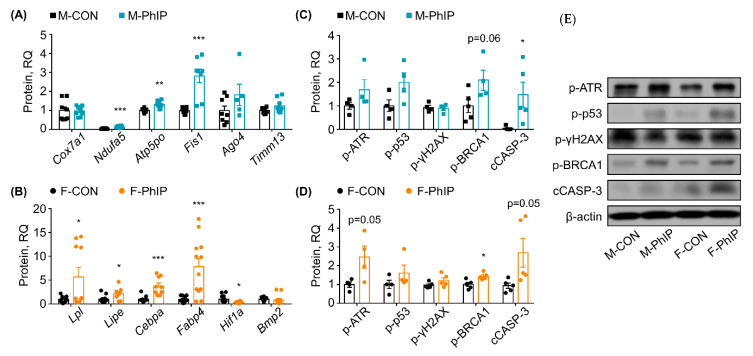
Validation of transcriptomics results using quantitative polymerase chain reaction (qPCR) analysis and assessment of protein expressions involved in DNA damage response (DDR) and apoptosis markers. (**A**) Genes were randomly selected from the top canonical pathways (i.e., Oxidative Phosphorylation, Mitochondria Dysfunction, eIF2 Signaling and Sirtuin Signaling Pathway) of the male colon transcriptome and assessed using qPCR analysis. (**B**) Genes were randomly selected from the top canonical pathways (i.e., Triacylglycerol Degradation, Retinol Biosynthesis and Adipogenesis Pathway) of the female colon transcriptome and assessed using qPCR analysis. Values are means ± SEM (*n* = 5, n for repeated measures = 7–8). (**C**,**D**) Key proteins responsible for the DDR signaling pathway and apoptosis markers were measured using (**E**) Western blot analysis. Representative protein bands for DDR signaling pathway and apoptosis marker proteins were quantified. Values are means ± SEM (*n* = 4–5). * *p* < 0.05, ** *p* < 0.01, *** *p* < 0.001 (examined by a two-tailed, Welch’s *t*-test using GraphPad Prism 3.0 software; GraphPad, San Diego, CA, USA). M-PhIP, male mice treated with PhIP; M-CON, control male mice; F-PhIP, female mice treated with PhIP; F-CON, control female mice.

Based on the IPA Canonical Pathway analysis, representative genes in the DEGs were selected and examined using quantitative real-time polymerase chain reaction (qPCR) analysis to validate the RNAseq results and bioinformatics predictions. For qPCR validation, random genes within selected canonical pathways were chosen rather than validating genes expressed the most or the least. The justification is twofold. In general, based on our previous experience, RNAseq provides overall great reproducibility for genes modified significantly. Second, the magnitude of changes in gene expression itself give little insight in terms of biological functions; in contrast, selection of genes following bioinformatics analysis (which is how we performed in the study) would be more informative for not only confirming experimental validity yet also interpretation of transcriptomic signatures in response to exposure to PhIP. In male mice, the following key genes involved in the predicted canonical pathways (i.e., OXPHOS and Mitochondrial Dysfunction) were assessed—Mitochondrial cytochrome c oxidase polypeptide 7a1 (*Cox7a1*), NADH dehydrogenase [ubiquinone] 1 alpha subcomplex subunit 5 (*Ndufa5*), mitochondrial ATP synthase subunit O (*Atp5po*) and mitochondrial fission 1 (*Fis1*). Expression levels of *Ndufa5*, *Atp5po* and *Fis1* were up-regulated compared to those of CON mice, while *Cox7a1* was not altered in the qPCR analysis (Figure 3A). Further, a few genes from the eIF2 Signaling and Sirtuin Signaling Pathway were also examined using qPCR analysis. Argonaute 4 (*Ago4*) and mitochondrial import inner membrane translocase subunit Tim13 (*Timm13*) mRNA expressions were not changed by PhIP treatment in male mice colon tissues (Figure 3A). In female mice, expressions of lipoprotein lipase (*Lpl*) and hormone-sensitive lipase (*Lipe*) associated with Triacylglycerol Degradation and Retinol Biosynthesis Pathway were up-regulated by PhIP treatment in colon tissues (Figure 3B). Moreover, expression of CCAAT/enhancer-binding protein alpha (*Cebpa*) and Fatty acid binding protein 4 (*Fabp4*), gene in the Adipogenesis Pathway, was significantly up-regulated (Figure 3B). Additional genes involved in the Adipogenesis Pathway, hypoxia-inducible factor 1-alpha (*Hif1a*) and bone morphogenetic protein 2 (*Bmp2*), were analyzed. *Hif1a* expression level was down-regulated by PhIP treatment consistently with the RNAseq results but changes in *Bmp2* expression were marginal and without statistical difference (Figure 3B). Overall, qPCR validations demonstrated consistent results with the RNAseq results.

### 2.5. Comparison of Sex-Dependent Effects of PhIP on DNA Damage Response and Apoptosis

Levels of key proteins involved in DNA damage response (DDR) and apoptosis were assessed using western blot analysis to briefly compare whether sex-dependent effects of PhIP influence the DDR signaling pathway and apoptosis in colon tissues (Figure 3C,D). There was no difference in phosphorylated ataxia telangiectasia and Rad3 related (p-ATR) protein level in PhIP-treated male mice (Figure 3C), but it was increased in female mice (Figure 3D). Neither sex showed altered expression of phosphorylated H2A histone family member X (p-γH2AX) and phosphorylated tumor protein p53 (p-p53) (i.e., active form) proteins by PhIP treatment. However, cleaved caspase-3 (cCASP-3) (an apoptotic maker) was significantly increased in PhIP-treated groups. Additionally, phosphorylated breast cancer type 1 (p-BRCA1) was increased in both male and female PhIP-treated mice.

## 3. Discussion

Sex differences are associated with cancer, specifically in incidence, prognosis and mortality [17]. Men show approximately 30% and 40% higher incidence and mortality rates of colorectal cancer, respectively, than those of women in the US [18]. In that regard, it is reasonable to expect that dietary carcinogens (including PhIP) may have sex-specific impacts. Here, we performed a comparative study to explore sex-dependent effects of PhIP on the colon transcriptome in mice. Molecular mechanisms regarding PhIP-induced colon carcinogenesis are well-established and documented [6,7,8,9]; therefore, rather than looking at the effects of sex on those pathways, we carried out an unbiased transcriptomics analysis which may provide insights as to novel signaling pathways impacted by PhIP. Our RNAseq results clearly showed different transcriptome signatures which were demonstrated in functional enrichment via GO analysis and major canonical pathways via IPA prediction.

All GO analyses, including Biological Processes, Molecular Functions and Cellular Compartments, evidently demonstrated that mitochondria-related processes and functions are significantly enriched in male mice (Table S4). Several genes related to the respiratory electron transport chain (e.g., *Cox1*, *Cox2*, *Cox5b*, *Cox7a1*, *Cox7c*, *Ndufa5* and *Ndufs6*) were affiliated with mitochondria-related biological processes. These findings are in line with the results from IPA pathway prediction; specifically, the IPA Canonical Pathway analysis predicted that OXPHOS was the most significantly influenced canonical metabolic pathway by PhIP in male mice (Figure 2A). Expression of key genes involved in the OXPHOS pathway, *Atp5po*, *Cox7a1* and *Ndufa5*, was confirmed using qPCR analysis (Figure 3A). In addition to the canonical metabolic pathway, canonical signaling pathways were also predicted using the IPA software; Mitochondrial Dysfunction was the most significant signaling pathway in male mice treated with PhIP, followed by eIF2 Signaling and Sirtuin Signaling Pathway (Figure 2B).

The OXPHOS metabolic pathway synthesizes ATP by electron transfer to a series of protein complexes in mitochondrial inner membranes. The OXPHOS pathway has been recognized as an anticancer target since down-regulated OXPHOS is manifested in cancers [19]. In fact, 13 subunits of OXPHOS protein complexes are coded by mitochondrial DNA (mtDNA) [20]; hence, there is likely a link between OXPHOS inactivation and mitochondrial dysfunctions such as mtDNA mutations. In the present study, however, OXPHOS was predicted to be activated in PhIP-treated male mice, which was confirmed by increased *Atp5po* and *Ndufa5* mRNA expressions (Figure 3A). These findings are still plausible as our animals did not develop colon cancer yet. Given that induction of colonic DNA adduct formation (i.e., early cancer marker) occurs 24 h after PhIP treatment and the adducts are generally repaired by a DNA repair system [21], the increase in OXPHOS might be a result from a compensatory response to excessive ATP consumption. An enormous ATP supply is required to account for a high ATP consumption rate that might be utilized for DNA repair [22,23]. In our study, we observed multiple terms enriched in the DEGs related to nucleotide repair as well as ATP biosynthesis (Supplementary Table S4). Further, a canonical signaling pathway prediction from the IPA Core Analysis provided supportive evidence of mitochondrial dysfunction, suggesting that over-consumption of ATP for DNA repair might induce OXPHOS activation, thereby causing the mitochondrial dysfunction. Further studies as to implications in colon carcinogenesis of PhIP in mitochondrial dysfunction, especially in the context of OXPHOS, are clearly warranted.

Metabolic signatures of the colon transcriptome in PhIP-treated female mice were completely different than those of male mice. In the female mice, carboxylic acid transport/metabolic processes are the highly enriched colonic biological processes; moreover, it was demonstrated that the following genes [annexin A1 (*Anxa1*), *Anxa3*, DDHD domain-containing protein 2 (*Ddhd2*), *Lpl*, carboxylic ester hydrolase 1g(*Ces1g*), *Ces2g*, *Ces1f* and *Lipe* in the DEGs] were related to the molecular function lipase activity. The IPA canonical metabolic pathway analysis denoted Triacylglycerol Degradation as a top metabolic pathway with a positive z-score (Figure 2C). *Ddhd2*, *Lpl* and *Lipe* were up-regulated in the pathway in PhIP-treated female mice and qPCR validation showed these same results (Figure 3B). Similarly, the Adipogenesis Pathway was predicted to be the third most significant canonical signaling pathway without an activity pattern predicted (Figure 2D). The qPCR validation analyses were consistent with the RNAseq results for *Cebpa* and *Fabp4* expressions. Interestingly, the results indicated that both the lipolysis and adipogenesis pathways were activated; key gene expression levels were all up-regulated in PhIP-treated female mice compared to CON mice (Figure 3B). Lipolysis is responsible for the catabolism of triacylglycerol caused by lipoprotein lipase (i.e., *Lpl* and *Lipe*) and subsequently releases free fatty acid to yield sufficient energy [24]. In addition, up-regulated *Lpl* and *Lipe* increase uptake of lipoproteins, which plays a role in hydrolysis of retinyl ester to synthesize retinol [25]. Adipogenesis can be controlled by retinol saturase, which catalyzes the conversion of retinol to dihydroretinoid. It has been reported that ablation of retinol saturase in preadipocytes inhibits adipogenesis [26], suggesting there might be an indirect regulatory role of PhIP treatment in adipogenesis because retinol biosynthesis was predicted to be induced in colon tissues from PhIP-treated female mice. In addition to *Cebpa* and *Fabp4*, other mRNAs in retinol biosynthesis were validated. *Hif1a* expression was decreased in PhIP-treated female mice but a marginal change was detected in *Bmp2* expression. Collectively, GO analysis, IPA Canonical Pathway prediction and qPCR validation suggest concurrent activation of adipogenesis and lipolysis when female mice were treated with PhIP.

An association between lipid metabolism and cancer has been reported [27]. Oncogenic signaling-mediated fatty acid synthesis and increased mobilization of fatty acids from adipose tissues lead lipids to be more available in cancer cells [28]. It subsequently promotes cancer development in different aspects such as cell growth and proliferation [29], resistance to oxidative stress [30] and stimulation of signaling pathways leading to proliferation and invasion [31]. Of note, it was found that a co-culture of adipocytes (originating from colon cancer patients and colon cancer cells) led to free fatty acid transfer from the adipocyte to the cancer cells [32]. The cancer cells survived in a nutrition-depleted environment via up-regulation of mitochondrial fatty acid β-oxidation resulting from lipolysis. This study hints that PhIP-induced adipogenesis in female mice may be preparing to provide more fatty acids available for cancerous cells to acquire energy from fatty acid β-oxidation via lipolysis. Another recent report demonstrated that lipolytic products such as macrophages initiate a local inflammatory reaction which, in turn, triggers beige adipogenesis via transient inflammation [33]. Therefore, lipid metabolism disruption in colon tissue might result from PhIP-induced inflammation. Taken together, PhIP-induced dysregulation of lipid metabolism in colon tissues of female mice can be multifactorial, hence requiring additional functional and mechanistic studies.

As aforementioned, PhIP causes DNA lesions by forming bulky adducts [12]. The bulky DNA adducts can be eliminated via the DDR signaling pathway [34]. Hence, we further examined a few key protein markers for the DDR signaling pathway as well as apoptosis. In a DDR signaling pathway, p-ATR is rapidly activated by bulky DNA adducts (e.g., PhIP adducts) and then p-p53 protein [35,36]. p53 is involved in recruitment of DNA repair proteins such as γH2AX [37] and is also able to trigger apoptosis depending upon the degree of DNA damages [38]. Overall, p-ATR was increased in PhIP-treated female mice while no difference was noted in the male mice. No change was noted in p-γH2AX and p-p53 protein in PhIP-treated groups, regardless of sex. However, cCASP-3 was significantly increased in PhIP-treated groups. Last, another important protein involved in DDR pathway (i.e., BRCA1) was assessed; BRCA1 is known to enhance nucleotide excision repair which is a p53-independent mechanism [39]. Likewise, p-BRCA1 was increased in both male and female PhIP-treated mice. Overall, except for p-ATR, we were not able to find a clear difference in DDR pathway/apoptosis-related markers between sexes which might be due to the short-term/single exposure to PhIP. In order to further validate our RNA-seq data and to explore impacts of signaling pathways that are differently enriched between sexes, additional studies with cancer phenotypes are warranted.

PhIP has been considered a mutagen and carcinogen in the colon and prostate of male rats [40] and in the mammary gland of female rats [41]. Further, sex-dependent responses to HAAs in carcinogenesis were previously reported using different rodent models [42]. The responses vary depending upon the duration of PhIP exposure and animal species. For instance, only male F344 rats developed tumors and DNA adducts in colon and liver tissues [15,43], suggesting male F344 rats are more susceptible than females. On the other hand, female CDF_1_ mice were more susceptible than male mice to lymphomas and hepatocarcinogenesis induced by chronic PhIP exposure [14]. There also exist reports on sex-dependent effects of PhIP on mutation of a specific gene. In the reports, a mutation frequency in a lac repressor gene in the kidney was significantly different in male and female rats after PhIP exposure [13]. No sex difference, however, was found in the mutation frequency of colonic lac repressor gene [44]. These former reports imply that PhIP effects can be both tissue-specific and sex-specific. However, there is still a lack of information as to what causes the sex-dependent responses to PhIP exposure. It is speculated that hormonal differences between male and female may cause the different responses to PhIP exposure. Knowing that CYPs, including CYP1A2, play important roles in estrogen metabolism by catalyzing estradiol 2-hydroxylation [45] as well as carcinogenesis [46], hormonal differences might somehow impact the effects of PhIP sex-dependently. For instance, CYPs (e.g., CYP1A2) catalyze 2-hydroxylation of estradiol in which the product (i.e., 2-methoxyestradiol) inhibits cancer cell proliferation [47,48], suggesting reduced CYP1A2 activity due to PhIP exposure may trigger carcinogenesis. Although estradiol plays a role in males as well, it is the predominant estrogen that is the primary female sex hormone. It was also suggested that androgen level contributes to susceptibility to liver cancer in male rats [49]. Moreover, androgenic activity of PhIP was reported due to a similar structure of PhIP to androgens [40], which might be related to the incidence of hormone-sensitive cancers in response to PhIP (e.g., prostate cancer). Collectively, further studies on hormone and colon cancer susceptibility crosstalk in response to PhIP exposure are warranted as a future research agenda.

In our experiment, mice were intraperitoneally injected with a single dose of PhIP (10 mg/kg body weight) as an acute exposure model; after, in order to characterize colonic transcriptome signatures using bioinformatics analyses, lists of DEGs were generated based on *p*-value < 0.05 and fold change ≥ 1.2. In this, two things should be noted as limitations. First, the acute exposure to PhIP is not sufficient to develop colon tumors (or related pathophysiological markers) in C57BL/6 mice. In fact, the mice strain is extremely resistant to developing colon tumors using PhIP, hence it requires specific genetic manipulation (e.g., humanized mice model) and/or co-exposure with another agent (e.g., dextran sodium sulfate). To be more specific, the reason that mice models (not only this C57BL/6) and rat models are resistant to developing colon tumor phenotype is due to their ability to attach a hydroxyl group at the 4′ position of PhIP rather than the *N*^2^ position in the liver. Regardless of the mouse/rat models, this characteristic remains the same, hence it is an inherent issue for all rodent models used for PhIP research. In other words, the resistance to developing colon tumors in rodents using PhIP is most likely not due to different responses in the colon, but in the liver. Despite its favored action on 4′ hydroxylation, activation of DDR and apoptotic responses suggest that an appreciable amount of *N*^2^ hydroxylated PhIP is indeed reached in the colon. As such, other studies, using rodent model, relied on either PhIP-related DDR or pre-cancerous lesions (e.g., aberrant crypt foci) to determine PhIP toxicity [50,51]. Therefore, before we further expand our study, it was a logical step for us to first explore if acute PhIP exposure is able to induce colonic transcriptome changes and if so, whether the signatures are sex-dependent. Second, the fold change, as a criterion, is arbitrary in nature. The rationale for the fold change (i.e., 1.2 fold change) was that we aimed to be inclusive especially considering the PhIP exposure condition which was acute and single dose. We do believe that ‘the inclusiveness’ was effective to determine the PhIP-induced colonic transcriptome signatures because with the genes selected based on 1.5 fold change, the GO analysis failed to enrich signaling in the PhIP-treated male mice. In contrast, in female mice, GO analysis showed that ‘Monocarboxylic acid transport,’ ‘Xenobiotic stimulus,’ ‘Fatty acid metabolic process,’ ‘Inflammatory response’ and ‘Cellular response to chemical stimulus’ were enriched which is somewhat similar to the results when genes were shortlisted with 1.2 fold change.

## 4. Materials and Methods

### 4.1. Animal Housing and Care

Animal handling and experiments were conducted according to a protocol approved by the Institutional Animal Care and Use Committee of the University of Arkansas (Protocol approval number: 19046; approved on 22 February 2019). Eight-week-old C57BL/6N mice (i.e., sexually reproductive age of mice) were purchased from the Jackson Laboratory (Bar Harbor, ME, USA) and housed in the Central Laboratory Animal Facility of the University of Arkansas. Upon delivery, the mice were acclimated in standard mice cages. Commercial semi-purified diet (AIN-93G) and filtered tap water were provided ad libitum during the acclimation period. Temperature (23 ± 2 °C), humidity (50 ± 5%) and a daily 12 h light-dark cycle were maintained.

### 4.2. Study Design and PhIP Treatment

A total of 20 mice (10 males and 10 females) were randomly assigned to four experimental groups—male control group (M-CON), male PhIP group (M-PhIP), female control group (F-CON) and female PhIP group (F-PhIP). After the acclimation period, mice in PhIP groups received an intraperitoneal injection of PhIP (10 mg/kg of body weight, dissolved in DMSO; Toronto Research Chemicals Inc., North York, ON, Canada). The route of exposure to PhIP was discussed and justified in our previous publication [12]. The injection volume of PhIP did not exceed 100 μL and was calculated based on body weight. Control group mice were treated with DMSO (10 mL/kg of body weight). Daily food intake was measured and behavioral activity was monitored to assess animal health and well-being after PhIP treatment. Twenty-four hours after the PhIP injection, the mice were euthanized by exposure to CO_2_ gas. Liver and colon tissues were harvested and stored at −80 °C in RNALater solution for further analyses.

### 4.3. Colon Transcriptomic Analysis

Total RNA from colon tissue was extracted using the RNeasy Plus Universal Mini Kit (Qiagen, Hilden, Germany) in accordance with the manufacturer’s protocol. The quality and quantity of RNA were assessed using SpectraMax i3x (Molecular Devices, Sunnyvale, CA, USA). In addition, the RNA Integrity Number (RIN) was assessed using the RNA R6K assay using the Agilent 220 TapeStation (Agilent Technology, Santa Clara, CA, USA). RNA samples with RIN above 7.0 showing two clear peaks for 18S and 28S RNAs were utilized for further RNAseq analysis (Table S6). Three samples per group were randomly selected and colon transcriptomics analysis was performed at the Research Technology Support Facility, Michigan State University (East Lansing, MI, USA) on the Illumina HiSeq 4000 system (1 × 50 bp single end read method, 4 sample indexing in a single lane of the flowcell, Illumina Inc., San Diego, CA, USA) as described in a previous report [52]. To normalize the library, the total read counts were transformed into the log_2_ number of reads per million to stabilize the variance. The normalized values were then subjected to further statistical analyses and computational bioinformatics analyses.

### 4.4. Bioinformatics Analyses

The log_2_ transformed transcriptomics dataset was utilized for statistical analysis and generation of a list of DEGs. In addition, fold change over each control group was calculated. Specifically, DEGs were first sorted based on our cut-off criteria—(1) mRNAs between CON and PhIP groups showed *p*-value < 0.05 and (2) fold change ≥ 1.2; by doing so, we were able to retrieve less than 1000 DEGs which is reasonable to handle for bioinformatics analyses. In the DEGs, genes in common between male and female datasets were excluded to compare sex-dependent effects of PhIP. The list of genes excluded from the DEGs was provided in Table S7. The data was then used to construct hierarchical clustering with a heatmap. Hierarchical clustering analysis was performed using the Pearson’s correlation coefficient and the average linkage method to group genes with similar expression patterns. The hierarchical clustering analysis and the heatmap between CON and PhIP groups in each sex were based on the log_2_ fold change values. All DEG data analysis, the clustering analysis and the heatmap generation were performed by R software package v. 3.6.1 (www.r-project.org).

In addition, over-represented (or under-represented) changes were assessed using the PANTHER over-repression test tool to identify gene enrichment associated with transitions between CON and PhIP groups. The DEGs were compared to a web-based reference list of PANTHER tools using the GO-terms (available at pantherdb.org). Furthermore, the DEGs were subjected to the IPA software in which Core Analysis was performed to predict related canonical pathways in response to PhIP treatment. Computational predictions using the IPA software were based on open source database of the associations of genes and related up/downstream genes archived by Ingenuity Knowledge Base.

### 4.5. Quantitative RT-PCR Analysis

The qPCR analysis was performed using StepOnePlus system (Applied Biosystems, Foster City, CA, USA). Five times diluted cDNA samples made up of two μg of total RNA were utilized as template DNAs, which was mixed with TaqMan Fast Advanced Master Mix and respective primers tagged with the PrimeTime qPCR probes (Table S8). The PCR amplification conditions were as follows—One cycle at 50 °C for 2 min and 95 °C for 2 min, followed by 40 cycles of denaturation (95 °C for 1 s) and annealing/extension (60 °C for 20 s). Expression levels of mRNA were normalized to a reference gene (Glyceraldehyde 3-phosphate dehydrogenase), which was the normalization control. Threshold cycles were automatically detected by StepOne Software (v. 2.1; Applied Biosystems) and data were represented as a relative quantity calculated using the 2^−ΔΔCt^ method.

### 4.6. Western Blot Analysis

Protein expression was measured by western blot analysis. In brief, total proteins were isolated from colon tissues and were prepared at a concentration of 1 mg/mL by diluting with the Laemmli buffer containing 10% sodium dodecyl sulfate and 5% 2-mercaptoethanol. Loaded proteins were separated via sodium dodecyl sulfate polyacrylamide gel electrophoresis and blotted to polyvinylidene fluoride membranes. After blocking the membranes using 5% bovine serum albumin in Tris-buffered saline (0.5 M Tris base, 9% NaCl and 2% Tween 20; pH 7.8), the membranes were incubated with the following primary antibodies for 12 h at 4 °C: β-actin, p-ATR, p-BRCA1, cCASP-3, p-γH2AX and p-p53 (Cell Signaling Technology, Danvers, MA, USA). Subsequently, the membranes were washed and then incubated with respective horseradish peroxidase-conjugated secondary antibodies for 1 hr at room temperature. Protein bands were visualized using the ChemiDoc Imaging System (Bio-Rad Laboratories, Hercules, CA, USA) and their intensity was quantified using ImageJ software (National Institutes of Health, Bethesda, MD, USA). Each membrane included a reference sample, which is used in all blots, and the final results were calculated as the ratio of protein/β-actin divided by the ratio of the reference sample/β-actin to factor in inter-assay variation.

### 4.7. Statistical Analyses

The qPCR data were analyzed by a two-tailed, Welch’s *t*-test using GraphPad Prism 3.0 software (GraphPad, San Diego, CA, USA). A *p*-value less than 0.05 was considered statistically significant. The *p*-values in PANTHER were adjusted using a Bonferroni correction within the tool and were calculated by Fisher’s exact test for the IPA.

## 5. Conclusions

In the present study, we comprehensively explored the sex-dependent effects of acute exposure to PhIP via unbiased transcriptomics analysis in mice colon. As discussed, male and female mice represented distinctive responses to PhIP treatment in terms of biological processes, molecular functions, cellular compartments and canonical pathways. In brief, in male mice, OXPHOS in the mitochondrial respiratory chain was the most significant pathway, which is thought to be resulting from a shortage of ATP for DNA repair, thereby causing mitochondrial dysfunction. However, results from female mice showed concurrent activation of lipolysis and adipogenesis that could increase risk for later stages of cancer development. This study provides foundations and directions for future studies of PhIP effects on sex-dependent mechanisms, which may merit following further topics—(1) comparing phenotypes and underlying mechanisms of PhIP-induced colon carcinogenesis between male and female (i.e., PhIP-DNA adduct), (2) conducting proper functional tests to confirm predicted pathways and mechanisms and (3) performing observational studies with colon cancer patients comparing sex differences.

## Figures and Tables

**Table 1:** List of genes involved in key canonical pathways predicted by the Ingenuity Pathway Analysis (IPA) software.

Canonical Pathways ^1^	Genes Up-Regulated	Genes Down-Regulated
M-PhIP vs. M-CON ^2^		
Oxidative Phosphorylation	*Atp5g1, Atp5po, Cox17, Cox6b1, Cox7a1, Cyb5a, Atp6, Ndufa2, Ndufa3, Ndufa5, Ndufa8, Ndufa11, Ndufb7, Ndufs6, Ndufs7, Ndufv3*	-
Mitochondrial Dysfunction	*Atp5g1, Atp5po, Cox17, Cox6b1, Cox7a1, Cyb5a, Fis1, Glrx2, Atp6, Ndufa2, Ndufa3, Ndufa5, Ndufa8, Ndufa11, Ndufb7, Ndufs6, Ndufs7, Ndufv3*	-
eIF2 Signaling	*Eif3k, Map2k2, Rpl18, Rpl24, Rpl28, Rpl35, Rpl38, Rpl27a, Rpl37a, Rplp2, Rps11, Rps19, Rps20, Rps26*	*Ago4, Eif2ak3, Pik3cb, Vegfa*
Sirtuin Signaling Pathway	*Atp5g1, Bax, Map1lc3a, Atp6, Ndufa2, Ndufa3, Ndufa5, Ndufa8, Ndufa11, Ndufb7, Ndufs6, Ndufs7, Ndufv3, Pam16, Sod1, Timm13, Tomm6*	*Acss2, Ppargc1a*
F-PhIP vs. F-CON		
Triacylglycerol Degradation	*Abhd6, Ddhd2, Lipe, Lpl*	*Aadac, Prdx6*
Retinol Biosynthesis	*Ces1g, Ddhd2, Lipe, Lpl*	*Aadac*
Adipogenesis Pathway	*Cebpa, Fabp4, Fgfrl1, Lpl, Sap30*	*Bmp2, Hif1a, Noct*

^1^ Short-listed differentially expressed genes were subjected to IPA Core Analysis to predict significant canonical pathways influenced by PhIP treatment; ^2^ M-PhIP, male mice treated with PhIP; M-CON, control male mice; F-PhIP, female mice treated with PhIP; F-CON, control female mice.

## Supplementary Materials

The following are available online at https://www.mdpi.com/1422-0067/21/18/6620/s1, Table S1: A list of differentially expressed genes in colonic transcriptomics dataset of male mice, Table S2: A list of differentially expressed genes in colonic transcriptomics dataset of female mice, Table S3: Significantly overrepresented Protein Annotation through Evolutionary Relationships gene ontology for colonic transcriptome of 2-amino-1-methyl-6-phenylimidazo[4,5-b]pyridine-treated male and female mice, Table S4: Significantly overrepresented Protein Annotation through Evolutionary Relationships (PANTHER) gene ontology (GO) terms for colonic transcriptome of 2-amino-1-methyl-6-phenylimidazo[4,5-b]pyridine (PhIP)-treated male mice, Table S5: Significantly overrepresented PANTHER gene ontology (GO) terms for colonic transcriptome of PhIP-treated female mice, Table S6: RNA Integrity Number (RIN) for colonic RNA samples used for transcriptomics, Table S7: A list of differentially expressed genes in common between male and female colonic transcriptomics dataset, Table S8: Primer information.

## Abbreviations

PhIP	2-amino-1-methyl-6-phenylimidazo[4,5-b]pyridine
Ago4	Argonaute 4
Anxa1	Annexin A1
Atp5po	Mitochondrial ATP synthase subunit O
Bmp2	Bone morphogenetic protein 2
cCASP-3	Cleaved caspase-3
Cebpa	CCAAT/enhancer-binding protein alpha
Ces	Carboxylic ester hydrolase
Cox7a1	Mitochondrial cytochrome c oxidase polypeptide 7a1
CYP	Cytochrome P450
Ddhd2	DDHD domain-containing protein 2
DDR	DNA damage response
DEGs	Differentially expressed genes
Fabp4	Fatty acid binding protein 4
F-CON	Female control group
Fis1	Mitochondrial fission 1
F-PhIP	Female PhIP group
GO	Gene ontology
HAAs	Heterocyclic aromatic amines
Hif1a	Hypoxia-inducible factor 1-alpha
IPA	Ingenuity pathway analysis
Lipe	Hormone-sensitive lipase
Lpl	Lipoprotein lipase
M-CON	Male control group
M-PhIP	Male PhIP group
mtDNA	Mitochondrial DNA
Ndufa5	NADH dehydrogenase [ubiquinone] 1 alpha subcomplex subunit 5
OXPHOS	Oxidative phosphorylation
PANTHER	Protein annotation through evolutionary relationships
p-ATR	Phosphorylated ataxia telangiectasia and Rad3 related
p-BRCA1	Phosphorylated breast cancer type 1
p-γH2AX	Phosphorylated H2A histone family member X
p-p53	Phosphorylated tumor protein p53
qPCR	Quantitative real-time PCR
RIN	RNA integrity number
RNAseq	RNA sequencing
Timm13	Mitochondrial import inner membrane translocase subunit Tim13
