# Supplementary material for: Colon Transcriptomics Reveals Sex-Dependent Metabolic Signatures in Response to 2-Amino-1-methyl-6-phenylimidazo[4,5-b]pyridine Treatment in C57BL/6N Mice
Source: Int J Mol Sci. 2020 Sep 10;21(18):6620. doi: 10.3390/ijms21186620 (PMC7555907; doi:10.3390/ijms21186620)
Supplement: Supplementary file 1 [file ijms-21-06620-s001.zip › Table S1.docx]

**Table S1.** A list of differentially expressed genes in colonic transcriptomics dataset of male mice.

| **Gene Symbol** | **Fold Change** | ***p*-Value** |
| --- | --- | --- |
| *Zfp597* | −4.660 | 0.012 |
| *Gxylt1* | −3.755 | 0.003 |
| *Elk4* | −3.745 | 0.045 |
| *Zbtb10* | −3.667 | 0.028 |
| *March9* | −3.323 | 0.027 |
| *Fam126b* | −2.941 | 0.040 |
| *LOC102640451* | −2.686 | 0.010 |
| *Mbnl3* | −2.681 | 0.027 |
| *Trdmt1* | −2.680 | 0.035 |
| *LOC108168164* | −2.614 | 0.037 |
| *Cacna2d2* | −2.492 | 0.026 |
| *6720489N17Rik* | −2.414 | 0.005 |
| *Nfkbie* | −2.344 | 0.035 |
| *Per3* | −2.294 | 0.049 |
| *Klhl28* | −2.259 | 0.032 |
| *Dusp8* | −2.198 | 0.007 |
| *Ppp1r3b* | −2.188 | 0.017 |
| *Gm29968* | −2.127 | 0.043 |
| *Gm29681* | −2.106 | 0.011 |
| *Zfp933* | −2.099 | 0.041 |
| *Foxn3* | −2.096 | 0.032 |
| *Wdr25* | −2.072 | 0.018 |
| *Gm39006* | −2.070 | 0.010 |
| *Cdc14b* | −2.042 | 0.032 |
| *Ago4* | −2.038 | 0.023 |
| *Ubn2* | −2.019 | 0.043 |
| *Gm38785* | −1.990 | 0.020 |
| *LOC108169155* | −1.975 | 0.007 |
| *Prdm15* | −1.975 | 0.027 |
| *Zfp280b* | −1.973 | 0.049 |
| *C1qtnf6* | −1.971 | 0.013 |
| *Tcaf2* | −1.971 | 0.040 |
| *Il18bp* | −1.964 | 0.034 |
| *Gm35150* | −1.963 | 0.006 |
| *Zdhhc17* | −1.956 | 0.035 |
| *Foxa2* | −1.946 | 0.007 |
| *Kcnf1* | −1.941 | 0.043 |
| *Mysm1* | −1.936 | 0.050 |
| *Trim72* | −1.914 | 0.039 |
| *Angel1* | −1.881 | 0.007 |
| *Zfp3* | −1.871 | 0.018 |
| *Fzd1* | −1.852 | 0.049 |
| *Leng8* | −1.821 | 0.015 |
| *Zkscan7* | −1.797 | 0.014 |
| *Kbtbd7* | −1.751 | 0.047 |
| *Cgnl1* | −1.742 | 0.047 |
| *Usp49* | −1.739 | 0.031 |
| *Stxbp1* | −1.737 | 0.045 |
| *Zfp799* | −1.736 | 0.028 |
| *LOC102633880* | −1.735 | 0.004 |
| *Smyd4* | −1.733 | 0.045 |
| *Nicn1* | −1.732 | 0.010 |
| *Gm28905* | −1.720 | 0.041 |
| *Phldb1* | −1.716 | 0.038 |
| *Map3k2* | −1.702 | 0.019 |
| *Trim59* | −1.693 | 0.011 |
| *A430033K04Rik* | −1.679 | 0.017 |
| *Tmem167b* | −1.661 | 0.030 |
| *Mpp7* | −1.655 | 0.022 |
| *Sgsm1* | −1.652 | 0.003 |
| *Zfp810* | −1.642 | 0.000 |
| *Cdk6* | −1.640 | 0.042 |
| *Cep83os* | −1.631 | 0.036 |
| *Slc46a1* | −1.628 | 0.015 |
| *Bbs4* | −1.622 | 0.000 |
| *Tbcel* | −1.621 | 0.014 |
| *Wipi1* | −1.612 | 0.041 |
| *Kcnk6* | −1.599 | 0.038 |
| *Gm11821* | −1.596 | 0.025 |
| *Selenbp2* | −1.584 | 0.040 |
| *Rcan3* | −1.578 | 0.016 |
| *Lnp* | −1.577 | 0.025 |
| *Gm30371* | −1.567 | 0.031 |
| *Ern1* | −1.564 | 0.018 |
| *Ccdc17* | −1.564 | 0.030 |
| *5330432E05Rik* | −1.560 | 0.016 |
| *Polh* | −1.559 | 0.023 |
| *Celsr1* | −1.558 | 0.036 |
| *Gm20544* | −1.548 | 0.009 |
| *Tbc1d12* | −1.547 | 0.030 |
| *Gm26596* | −1.545 | 0.036 |
| *Lyz2* | −1.544 | 0.047 |
| *Plch1* | −1.540 | 0.026 |
| *Mrm1* | −1.534 | 0.029 |
| *Rbm43* | −1.530 | 0.005 |
| *Pan3* | −1.528 | 0.046 |
| *2310039L15Rik* | −1.519 | 0.005 |
| *Glce* | −1.518 | 0.031 |
| *1810026J23Rik* | −1.517 | 0.030 |
| *4921536K21Rik* | −1.517 | 0.044 |
| *Pus7l* | −1.516 | 0.028 |
| *Panct2* | −1.505 | 0.036 |
| *Nol3* | −1.498 | 0.032 |
| *Bbs7* | −1.490 | 0.036 |
| *Gm6410* | −1.490 | 0.036 |
| *Herc3* | −1.487 | 0.010 |
| *Snhg11* | −1.486 | 0.039 |
| *Stx16* | −1.480 | 0.028 |
| *3110043O21Rik* | −1.477 | 0.004 |
| *8030462N17Rik* | −1.475 | 0.024 |
| *Lipt1* | −1.468 | 0.047 |
| *Ocrl* | −1.468 | 0.044 |
| *Fam35a* | −1.462 | 0.036 |
| *Grhl2* | −1.460 | 0.012 |
| *BC021891* | −1.460 | 0.040 |
| *4930427A07Rik* | −1.457 | 0.024 |
| *Tbc1d32* | −1.455 | 0.012 |
| *Gata6* | −1.451 | 0.031 |
| *Rab27b* | −1.451 | 0.032 |
| *Ccnt2* | −1.448 | 0.023 |
| *Fer1l6* | −1.444 | 0.002 |
| *Vps13c* | −1.437 | 0.048 |
| *Lmbrd2* | −1.434 | 0.018 |
| *Rnf38* | −1.433 | 0.032 |
| *Usp45* | −1.431 | 0.034 |
| *Shroom2* | −1.422 | 0.033 |
| *Vegfa* | −1.421 | 0.002 |
| *Rbm33* | −1.418 | 0.031 |
| *Zfat* | −1.418 | 0.019 |
| *Gm35829* | −1.415 | 0.042 |
| *Gm30400* | −1.414 | 0.039 |
| *Pm20d1* | −1.414 | 0.042 |
| *Gm31728* | −1.414 | 0.040 |
| *Pyroxd2* | −1.410 | 0.047 |
| *Ttc14* | −1.409 | 0.013 |
| *Mob3c* | −1.408 | 0.004 |
| *Egfr* | −1.407 | 0.034 |
| *6430548M08Rik* | −1.404 | 0.007 |
| *Alg6* | −1.401 | 0.047 |
| *Zfp276* | −1.399 | 0.030 |
| *Usp30* | −1.391 | 0.031 |
| *Gm41254* | −1.389 | 0.021 |
| *Gm36195* | −1.388 | 0.031 |
| *Map2k6* | −1.387 | 0.011 |
| *Muc4* | −1.387 | 0.027 |
| *Gm31566* | −1.385 | 0.017 |
| *Zfp644* | −1.379 | 0.046 |
| *Cwc25* | −1.374 | 0.041 |
| *Gm19950* | −1.374 | 0.044 |
| *Zfp251* | −1.372 | 0.045 |
| *Flt3l* | −1.368 | 0.043 |
| *Xylt2* | −1.366 | 0.041 |
| *Tmem245* | −1.364 | 0.004 |
| *Zbtb42* | −1.364 | 0.020 |
| *Lancl2* | −1.363 | 0.042 |
| *Atxn3* | −1.362 | 0.011 |
| *Arsb* | −1.360 | 0.034 |
| *Csgalnact2* | −1.360 | 0.034 |
| *Med20* | −1.359 | 0.024 |
| *Gm34589* | −1.357 | 0.029 |
| *Sesn3* | −1.357 | 0.045 |
| *Cdk19* | −1.353 | 0.042 |
| *D230025D16Rik* | −1.350 | 0.039 |
| *Swsap1* | −1.349 | 0.001 |
| *Epb41* | −1.348 | 0.032 |
| *Ranbp6* | −1.347 | 0.041 |
| *Gopc* | −1.347 | 0.021 |
| *Fam107b* | −1.346 | 0.005 |
| *Chm* | −1.345 | 0.045 |
| *Zfp160* | −1.342 | 0.031 |
| *Mid2* | −1.341 | 0.012 |
| *Mmp28* | −1.339 | 0.024 |
| *Otud4* | −1.336 | 0.030 |
| *Mlxip* | −1.335 | 0.044 |
| *Slc35b4* | −1.335 | 0.038 |
| *Entpd7* | −1.334 | 0.036 |
| *Rapgefl1* | −1.334 | 0.006 |
| *Naip6* | −1.333 | 0.031 |
| *H2−M3* | −1.330 | 0.001 |
| *D430042O09Rik* | −1.329 | 0.016 |
| *Col4a3bp* | −1.329 | 0.017 |
| *LOC108167680* | −1.326 | 0.038 |
| *Zfp280d* | −1.324 | 0.042 |
| *Zfp935* | −1.324 | 0.033 |
| *Hmg20a* | −1.321 | 0.047 |
| *Cd99l2* | −1.318 | 0.030 |
| *Exoc2* | −1.316 | 0.039 |
| *Cpd* | −1.315 | 0.011 |
| *Gm36556* | −1.312 | 0.048 |
| *Zfp748* | −1.312 | 0.015 |
| *Ap4m1* | −1.311 | 0.004 |
| *Wdr13* | −1.310 | 0.028 |
| *Rbmx2* | −1.309 | 0.031 |
| *Zfp715* | −1.309 | 0.009 |
| *A630020A06* | −1.308 | 0.031 |
| *Rgl3* | −1.308 | 0.032 |
| *Fam117b* | −1.307 | 0.001 |
| *Ntn4* | −1.307 | 0.015 |
| *Lama3* | −1.307 | 0.014 |
| *Agps* | −1.305 | 0.016 |
| *Ogt* | −1.302 | 0.042 |
| *Arhgap26* | −1.301 | 0.041 |
| *Zfp784* | −1.296 | 0.045 |
| *Tlr4* | −1.295 | 0.034 |
| *Zfp36l2* | −1.294 | 0.023 |
| *Gm40243* | −1.294 | 0.018 |
| *Zbtb2* | −1.292 | 0.020 |
| *Lrsam1* | −1.291 | 0.003 |
| *Gpatch2l* | −1.291 | 0.032 |
| *Cnpy4* | −1.291 | 0.002 |
| *Tox3* | −1.290 | 0.004 |
| *Scarb1* | −1.287 | 0.001 |
| *Klhl25* | −1.286 | 0.020 |
| *4932416H05Rik* | −1.285 | 0.007 |
| *Pdcl* | −1.285 | 0.043 |
| *Rpusd1* | −1.284 | 0.017 |
| *Desi2* | −1.280 | 0.002 |
| *Gm21811* | −1.279 | 0.012 |
| *Dcaf7* | −1.278 | 0.035 |
| *Trim36* | −1.275 | 0.008 |
| *Gm31862* | −1.275 | 0.012 |
| *Rab27a* | −1.274 | 0.038 |
| *Mknk1* | −1.274 | 0.040 |
| *Fabp5* | −1.273 | 0.044 |
| *LOC108167849* | −1.273 | 0.011 |
| *Foxa1* | −1.271 | 0.004 |
| *Cops7b* | −1.270 | 0.037 |
| *Sept8* | −1.270 | 0.001 |
| *Jrkl* | −1.269 | 0.027 |
| *Edem3* | −1.268 | 0.034 |
| *Prpf4b* | −1.268 | 0.035 |
| *Bin1* | −1.268 | 0.046 |
| *Ccdc15* | −1.267 | 0.009 |
| *Lamc2* | −1.266 | 0.004 |
| *Heatr6* | −1.264 | 0.043 |
| *Zfp800* | −1.262 | 0.016 |
| *Ppargc1a* | −1.262 | 0.042 |
| *Tbc1d23* | −1.261 | 0.017 |
| *Pde8a* | −1.259 | 0.011 |
| *Zbtb9* | −1.259 | 0.017 |
| *Aebp2* | −1.258 | 0.050 |
| *Gm34156* | −1.258 | 0.030 |
| *3110052M02Rik* | −1.257 | 0.048 |
| *Gm16041* | −1.257 | 0.033 |
| *Gcc1* | −1.257 | 0.022 |
| *Ganc* | −1.256 | 0.027 |
| *Elp4* | −1.256 | 0.036 |
| *Frmd4b* | −1.255 | 0.022 |
| *Tbc1d8b* | −1.254 | 0.030 |
| *Pik3cb* | −1.252 | 0.002 |
| *Vps33b* | −1.251 | 0.038 |
| *Rdh13* | −1.250 | 0.026 |
| *Pspc1* | −1.248 | 0.000 |
| *Usp28* | −1.247 | 0.039 |
| *Tmem168* | −1.247 | 0.035 |
| *Zfp260* | −1.246 | 0.021 |
| *Napg* | −1.245 | 0.005 |
| *Mbp* | −1.245 | 0.001 |
| *Fgd3* | −1.244 | 0.023 |
| *Galnt7* | −1.239 | 0.009 |
| *Eif2ak3* | −1.239 | 0.007 |
| *Eya3* | −1.238 | 0.020 |
| *Gnpda1* | −1.238 | 0.021 |
| *Clec14a* | −1.237 | 0.036 |
| *Tab1* | −1.236 | 0.001 |
| *Ivd* | −1.236 | 0.035 |
| *Gtf3c3* | −1.235 | 0.032 |
| *Apaf1* | −1.234 | 0.036 |
| *Rapgef6* | −1.234 | 0.005 |
| *Simc1* | −1.234 | 0.004 |
| *Mecom* | −1.233 | 0.018 |
| *Pcid2* | −1.233 | 0.016 |
| *Dennd6a* | −1.233 | 0.038 |
| *Acss2* | −1.232 | 0.008 |
| *Gm39257* | −1.232 | 0.021 |
| *Fnip1* | −1.232 | 0.037 |
| *Gsdmc3* | −1.230 | 0.034 |
| *6530403H02Rik* | −1.230 | 0.045 |
| *Cacfd1* | −1.229 | 0.019 |
| *C330007P06Rik* | −1.229 | 0.009 |
| *Ppargc1b* | −1.226 | 0.048 |
| *Xpr1* | −1.224 | 0.046 |
| *Appl2* | −1.223 | 0.010 |
| *Casc3* | −1.223 | 0.030 |
| *Dbt* | −1.222 | 0.017 |
| *Map4k5* | −1.221 | 0.032 |
| *Sh3bgrl2* | −1.221 | 0.007 |
| *Pcmtd2* | −1.220 | 0.035 |
| *Enpp5* | −1.220 | 0.001 |
| *Usf3* | −1.220 | 0.050 |
| *9130019O22Rik* | −1.219 | 0.023 |
| *Tecpr1* | −1.218 | 0.038 |
| *Gtf3c5* | −1.218 | 0.024 |
| *Gzf1* | −1.216 | 0.022 |
| *Rin2* | −1.216 | 0.022 |
| *Trim11* | −1.214 | 0.038 |
| *Wdsub1* | −1.213 | 0.002 |
| *Cachd1* | −1.213 | 0.013 |
| *Ankmy2* | −1.213 | 0.006 |
| *Foxd2* | −1.212 | 0.008 |
| *Far1* | −1.211 | 0.020 |
| *9130011E15Rik* | −1.210 | 0.034 |
| *Zxdc* | −1.210 | 0.014 |
| *Rdh11* | −1.209 | 0.013 |
| *Gm16070* | −1.209 | 0.007 |
| *Fnbp1l* | −1.208 | 0.016 |
| *Letmd1* | −1.207 | 0.028 |
| *Cpm* | −1.207 | 0.035 |
| *Pfkfb2* | −1.205 | 0.014 |
| *Hsd3b7* | −1.205 | 0.024 |
| *Ufl1* | −1.205 | 0.011 |
| *Pde7a* | −1.204 | 0.033 |
| *Itsn2* | −1.204 | 0.047 |
| *Aplf* | −1.203 | 0.024 |
| *Ighmbp2* | −1.203 | 0.012 |
| *Ndufs7* | 1.201 | 0.010 |
| *Tsfm* | 1.201 | 0.011 |
| *Eif3k* | 1.201 | 0.043 |
| *Coq7* | 1.202 | 0.025 |
| *Psme1* | 1.203 | 0.048 |
| *Cyb5a* | 1.204 | 0.017 |
| *Sf3b5* | 1.204 | 0.029 |
| *Slc39a5* | 1.204 | 0.027 |
| *LOC108169038* | 1.204 | 0.050 |
| *Srsf9* | 1.205 | 0.006 |
| *Uba52* | 1.205 | 0.050 |
| *9330136K24Rik* | 1.206 | 0.021 |
| *Pttg1* | 1.206 | 0.045 |
| *Pgs1* | 1.207 | 0.008 |
| *Rpl28* | 1.207 | 0.011 |
| *Ccdc124* | 1.208 | 0.010 |
| *COX1* | 1.209 | 0.007 |
| *Srek1ip1* | 1.209 | 0.019 |
| *Gm36227* | 1.209 | 0.018 |
| *Cd302* | 1.209 | 0.026 |
| *Dnaja1* | 1.210 | 0.002 |
| *Tmem179b* | 1.210 | 0.033 |
| *Rpl18* | 1.211 | 0.047 |
| *Degs2* | 1.213 | 0.029 |
| *Mrpl42* | 1.213 | 0.047 |
| *Ces1d* | 1.214 | 0.026 |
| *Gm32940* | 1.214 | 0.035 |
| *Rpl27a* | 1.214 | 0.047 |
| *Cisd3* | 1.214 | 0.004 |
| *Rbm8a* | 1.215 | 0.014 |
| *Sumo3* | 1.216 | 0.043 |
| *Alad* | 1.216 | 0.029 |
| *Hacd1* | 1.216 | 0.038 |
| *Rmdn3* | 1.217 | 0.023 |
| *Cyp4f13* | 1.218 | 0.043 |
| *Naprt* | 1.219 | 0.030 |
| *Csnk2b* | 1.219 | 0.035 |
| *Rps20* | 1.220 | 0.029 |
| *Prelid1* | 1.220 | 0.043 |
| *Gm9780* | 1.221 | 0.029 |
| *COX2* | 1.221 | 0.002 |
| *Map2k2* | 1.221 | 0.042 |
| *Gm4262* | 1.221 | 0.010 |
| *Rps26* | 1.221 | 0.020 |
| *ND1* | 1.222 | 0.002 |
| *Rad9b* | 1.223 | 0.006 |
| *Iscu* | 1.223 | 0.016 |
| *1700021F05Rik* | 1.223 | 0.024 |
| *Spint2* | 1.224 | 0.044 |
| *Zfp622* | 1.224 | 0.041 |
| *Cdk2ap2* | 1.225 | 0.030 |
| *Gm14204* | 1.225 | 0.038 |
| *Rpl38* | 1.225 | 0.016 |
| *Ptges3* | 1.225 | 0.037 |
| *1110018N20Rik* | 1.227 | 0.007 |
| *Ubxn1* | 1.227 | 0.041 |
| *2700097O09Rik* | 1.227 | 0.006 |
| *Ube2m* | 1.229 | 0.031 |
| *Akr7a5* | 1.232 | 0.029 |
| *Ftl1* | 1.235 | 0.030 |
| *Mrps34* | 1.236 | 0.033 |
| *Ccdc85b* | 1.236 | 0.014 |
| *Zdhhc12* | 1.236 | 0.029 |
| *Mrps18a* | 1.236 | 0.039 |
| *Map1lc3a* | 1.237 | 0.040 |
| *2810428I15Rik* | 1.238 | 0.038 |
| *Mrpl23* | 1.239 | 0.023 |
| *ATP6* | 1.239 | 0.018 |
| *Med9* | 1.240 | 0.040 |
| *Phpt1* | 1.242 | 0.035 |
| *Sepw1* | 1.242 | 0.027 |
| *Nt5m* | 1.242 | 0.015 |
| *Srrd* | 1.242 | 0.011 |
| *Cox6b1* | 1.243 | 0.045 |
| *Ppil1* | 1.243 | 0.050 |
| *Gm21885* | 1.243 | 0.042 |
| *Thap7* | 1.244 | 0.022 |
| *Cwh43* | 1.244 | 0.024 |
| *Dgat1* | 1.244 | 0.033 |
| *Ndufv3* | 1.244 | 0.037 |
| *Sod1* | 1.245 | 0.049 |
| *Kansl2* | 1.245 | 0.007 |
| *Snrpd3* | 1.247 | 0.049 |
| *Eci3* | 1.249 | 0.017 |
| *Dynlrb1* | 1.250 | 0.014 |
| *1110059G10Rik* | 1.250 | 0.022 |
| *Banf1* | 1.250 | 0.007 |
| *LOC108167554* | 1.251 | 0.015 |
| *Fam178b* | 1.252 | 0.036 |
| *Rps11* | 1.253 | 0.046 |
| *Atp5g1* | 1.254 | 0.039 |
| *BC004004* | 1.254 | 0.030 |
| *Pfdn6* | 1.256 | 0.028 |
| *Ccdc12* | 1.256 | 0.019 |
| *Tmem54* | 1.256 | 0.049 |
| *Tmem41a* | 1.257 | 0.013 |
| *ND6* | 1.257 | 0.003 |
| *1110038F14Rik* | 1.257 | 0.025 |
| *Snx3* | 1.258 | 0.039 |
| *LOC102640468* | 1.259 | 0.029 |
| *Swi5* | 1.259 | 0.046 |
| *Gm40474* | 1.259 | 0.003 |
| *Txnl4a* | 1.259 | 0.033 |
| *Rpl35* | 1.260 | 0.031 |
| *Lamtor2* | 1.260 | 0.029 |
| *Khk* | 1.260 | 0.032 |
| *Mvb12a* | 1.261 | 0.040 |
| *Rtca* | 1.262 | 0.039 |
| *Fgfbp1* | 1.262 | 0.044 |
| *Exosc4* | 1.264 | 0.009 |
| *Tmed1* | 1.265 | 0.042 |
| *Tmsb10* | 1.267 | 0.048 |
| *Tmem258* | 1.270 | 0.049 |
| *Pomp* | 1.270 | 0.049 |
| *2410004B18Rik* | 1.272 | 0.033 |
| *COX3* | 1.272 | 0.031 |
| *Sap18* | 1.273 | 0.019 |
| *Sap18b* | 1.273 | 0.019 |
| *Rpl24* | 1.273 | 0.038 |
| *Anapc13* | 1.273 | 0.017 |
| *Nenf* | 1.273 | 0.035 |
| *Rpl36* | 1.274 | 0.039 |
| *Rpp21* | 1.274 | 0.032 |
| *Atp5po* | 1.275 | 0.048 |
| *Rpl37a* | 1.276 | 0.036 |
| *Ndufa11* | 1.276 | 0.048 |
| *Mrpl30* | 1.276 | 0.027 |
| *Ap2s1* | 1.276 | 0.030 |
| *Nedd8* | 1.277 | 0.041 |
| *LOC108168260* | 1.278 | 0.025 |
| *Mzt2* | 1.278 | 0.036 |
| *Sp3os* | 1.279 | 0.012 |
| *H2−Q7* | 1.279 | 0.023 |
| *Chchd10* | 1.279 | 0.022 |
| *Bax* | 1.279 | 0.046 |
| *Rps19* | 1.281 | 0.034 |
| *Plekhj1* | 1.281 | 0.043 |
| *Agpat2* | 1.282 | 0.034 |
| *Cuta* | 1.283 | 0.004 |
| *Rplp2* | 1.284 | 0.027 |
| *Amdhd2* | 1.287 | 0.034 |
| *Zmat5* | 1.287 | 0.039 |
| *Gm10499* | 1.287 | 0.033 |
| *Ndufa8* | 1.287 | 0.020 |
| *Mrps21* | 1.288 | 0.032 |
| *Gm33729* | 1.289 | 0.006 |
| *Tmem223* | 1.289 | 0.045 |
| *Dleu2* | 1.289 | 0.021 |
| *Gm33232* | 1.289 | 0.008 |
| *Mettl7b* | 1.289 | 0.038 |
| *H2afv* | 1.290 | 0.026 |
| *Calml4* | 1.290 | 0.037 |
| *Glrx5* | 1.291 | 0.030 |
| *Grcc10* | 1.291 | 0.033 |
| *Edf1* | 1.291 | 0.040 |
| *Zcrb1* | 1.292 | 0.043 |
| *D8Ertd738e* | 1.292 | 0.025 |
| *Pam16* | 1.293 | 0.043 |
| *Sptssa* | 1.293 | 0.049 |
| *Gng5* | 1.293 | 0.042 |
| *Gm42081* | 1.296 | 0.048 |
| *Psmb9* | 1.297 | 0.047 |
| *Gadd45gip1* | 1.298 | 0.038 |
| *Cnbd2* | 1.298 | 0.009 |
| *Rpl29* | 1.299 | 0.045 |
| *Ndufa3* | 1.301 | 0.024 |
| *Fis1* | 1.301 | 0.045 |
| *Gm15645* | 1.302 | 0.018 |
| *1810058I24Rik* | 1.304 | 0.032 |
| *Isoc2a* | 1.304 | 0.031 |
| *Pde9a* | 1.305 | 0.024 |
| *Mrps12* | 1.306 | 0.015 |
| *Fth1* | 1.308 | 0.026 |
| *Cox5b* | 1.308 | 0.037 |
| *Sssca1* | 1.308 | 0.010 |
| *Psme2* | 1.309 | 0.036 |
| *Psmg3* | 1.309 | 0.042 |
| *Gstp1* | 1.312 | 0.015 |
| *LOC108167611* | 1.313 | 0.042 |
| *1810009A15Rik* | 1.314 | 0.015 |
| *Idnk* | 1.314 | 0.045 |
| *Romo1* | 1.315 | 0.027 |
| *Mrpl15* | 1.317 | 0.042 |
| *Mrpl55* | 1.318 | 0.041 |
| *Gstk1* | 1.320 | 0.024 |
| *LOC108167450* | 1.320 | 0.011 |
| *LOC108167641* | 1.322 | 0.025 |
| *Gm26744* | 1.323 | 0.038 |
| *LOC102639762* | 1.323 | 0.050 |
| *Ndufs6* | 1.327 | 0.038 |
| *Pdcd5* | 1.327 | 0.040 |
| *Cyp2c55* | 1.327 | 0.046 |
| *Snf8* | 1.327 | 0.036 |
| *Chchd7* | 1.327 | 0.048 |
| *Rnasek* | 1.327 | 0.029 |
| *Tmc4−ps* | 1.328 | 0.038 |
| *Znhit1* | 1.328 | 0.033 |
| *TrnS1* | 1.329 | 0.031 |
| *Taf10* | 1.330 | 0.024 |
| *LOC108167801* | 1.332 | 0.002 |
| *Mrps36* | 1.332 | 0.050 |
| *Gm15541* | 1.333 | 0.026 |
| *Znhit2* | 1.333 | 0.007 |
| *Atp5k* | 1.335 | 0.049 |
| *Ndufc2* | 1.336 | 0.049 |
| *Ndufb7* | 1.338 | 0.023 |
| *Tceb2* | 1.340 | 0.040 |
| *Smdt1* | 1.341 | 0.025 |
| *Gm32999* | 1.342 | 0.041 |
| *Pmm1* | 1.343 | 0.032 |
| *Cox17* | 1.347 | 0.049 |
| *Mrpl22* | 1.349 | 0.041 |
| *Rpusd3* | 1.350 | 0.024 |
| *Polr2f* | 1.351 | 0.010 |
| *Rad9a* | 1.351 | 0.034 |
| *Cox7c* | 1.351 | 0.048 |
| *Pnkd* | 1.353 | 0.049 |
| *Gm3718* | 1.353 | 0.038 |
| *Scand1* | 1.354 | 0.030 |
| *Bola1* | 1.354 | 0.049 |
| *Pgp* | 1.355 | 0.049 |
| *Snhg3* | 1.355 | 0.041 |
| *Gm35778* | 1.355 | 0.048 |
| *Med29* | 1.359 | 0.028 |
| *Gm10033* | 1.359 | 0.009 |
| *Mrpl34* | 1.360 | 0.016 |
| *Ndufa2* | 1.361 | 0.033 |
| *Ydjc* | 1.361 | 0.046 |
| *0610012G03Rik* | 1.365 | 0.045 |
| *Rps19bp1* | 1.365 | 0.038 |
| *Tceb1* | 1.366 | 0.044 |
| *mt−Rnr1* | 1.368 | 0.029 |
| *Exosc5* | 1.369 | 0.027 |
| *Ndufc1* | 1.370 | 0.029 |
| *Rab4a* | 1.371 | 0.031 |
| *Akr1b7* | 1.375 | 0.044 |
| *Ccdc58* | 1.376 | 0.032 |
| *Pin4* | 1.381 | 0.016 |
| *ND3* | 1.383 | 0.030 |
| *Gm14005* | 1.383 | 0.007 |
| *Iah1* | 1.384 | 0.047 |
| *Tomm6* | 1.385 | 0.005 |
| *Tmem29* | 1.385 | 0.016 |
| *Anapc11* | 1.387 | 0.035 |
| *Gm38907* | 1.390 | 0.036 |
| *Fxn* | 1.392 | 0.021 |
| *Gm32566* | 1.392 | 0.001 |
| *Ndufa5* | 1.394 | 0.039 |
| *Akr1c19* | 1.398 | 0.041 |
| *Gm12188* | 1.402 | 0.004 |
| *H2afj* | 1.402 | 0.046 |
| *Hsd17b2* | 1.403 | 0.036 |
| *Adarb2* | 1.404 | 0.040 |
| *Pdcd2* | 1.404 | 0.044 |
| *Gm12248* | 1.407 | 0.015 |
| *LOC102639758* | 1.407 | 0.023 |
| *Timm13* | 1.407 | 0.033 |
| *Hint2* | 1.408 | 0.043 |
| *Glrx2* | 1.409 | 0.008 |
| *Gm38676* | 1.409 | 0.015 |
| *Gm29376* | 1.414 | 0.036 |
| *Car1* | 1.420 | 0.042 |
| *Slirp* | 1.423 | 0.043 |
| *9430016H08Rik* | 1.424 | 0.046 |
| *Comtd1* | 1.424 | 0.045 |
| *Smim22* | 1.427 | 0.008 |
| *Mycbp* | 1.428 | 0.031 |
| *Ppp1r35* | 1.428 | 0.026 |
| *Higd1a* | 1.433 | 0.049 |
| *Spink4* | 1.433 | 0.029 |
| *Arpp19* | 1.434 | 0.036 |
| *A330069E16Rik* | 1.440 | 0.012 |
| *Coq3* | 1.444 | 0.049 |
| *Gm3837* | 1.451 | 0.046 |
| *Dgcr6* | 1.456 | 0.028 |
| *LOC105246506* | 1.458 | 0.039 |
| *Fam195a* | 1.460 | 0.041 |
| *2200002D01Rik* | 1.461 | 0.018 |
| *Smim8* | 1.462 | 0.037 |
| *Abhd11os* | 1.464 | 0.026 |
| *AI413582* | 1.467 | 0.025 |
| *Gm12618* | 1.468 | 0.048 |
| *Naa38* | 1.472 | 0.034 |
| *Bola3* | 1.473 | 0.024 |
| *Golt1a* | 1.478 | 0.047 |
| *1810043H04Rik* | 1.482 | 0.031 |
| *Ppdpf* | 1.484 | 0.019 |
| *Hdhd3* | 1.489 | 0.045 |
| *2310009A05Rik* | 1.495 | 0.041 |
| *Gm6745* | 1.505 | 0.034 |
| *Ly96* | 1.508 | 0.019 |
| *2010010A06Rik* | 1.510 | 0.043 |
| *LOC102637947* | 1.514 | 0.021 |
| *Tmem102* | 1.514 | 0.012 |
| *Gm4997* | 1.519 | 0.040 |
| *TrnE* | 1.521 | 0.012 |
| *Alkbh7* | 1.522 | 0.034 |
| *Rbks* | 1.524 | 0.026 |
| *Ttc32* | 1.524 | 0.035 |
| *Fam162a* | 1.544 | 0.020 |
| *Psmg4* | 1.549 | 0.049 |
| *Pnp2* | 1.557 | 0.045 |
| *Tmem261* | 1.559 | 0.012 |
| *Mrpl54* | 1.560 | 0.042 |
| *Sdhaf4* | 1.563 | 0.036 |
| *Bri3* | 1.569 | 0.027 |
| *Samd15* | 1.570 | 0.026 |
| *Snhg6* | 1.587 | 0.018 |
| *Gm5601* | 1.603 | 0.048 |
| *Gm12907* | 1.611 | 0.041 |
| *TrnC* | 1.623 | 0.038 |
| *Gm31532* | 1.644 | 0.031 |
| *Cox7a1* | 1.652 | 0.047 |
| *LOC102638797* | 1.667 | 0.042 |
| *Mrpl33* | 1.698 | 0.018 |
| *Gm34159* | 1.725 | 0.006 |
| *Cbr3* | 1.731 | 0.030 |
| *Gm32683* | 1.749 | 0.028 |
| *Hist2h2ac* | 1.764 | 0.043 |
| *BC039771* | 1.768 | 0.030 |
| *TrnY* | 1.782 | 0.033 |
| *Gm31214* | 1.784 | 0.035 |
| *Tctex1d2* | 1.847 | 0.016 |
| *Gm15506* | 1.856 | 0.042 |
| *Gm16150* | 1.857 | 0.029 |
| *Gsta1* | 1.887 | 0.036 |
| *3110009M11Rik* | 1.897 | 0.008 |
| *Gm30198* | 1.904 | 0.001 |
| *Lyrm2* | 1.929 | 0.009 |
| *2310009B15Rik* | 1.953 | 0.026 |
| *Gm30141* | 1.955 | 0.011 |
| *LOC108168984* | 1.958 | 0.025 |
| *Inmt* | 2.059 | 0.028 |
| *Gm30582* | 2.172 | 0.010 |
| *Gm40218* | 2.187 | 0.019 |
| *4930415O20Rik* | 2.223 | 0.043 |
| *Bc1* | 2.228 | 0.043 |
| *C86187* | 2.287 | 0.005 |
| *1700037C18Rik* | 2.418 | 0.044 |
| *Hist2h2aa1* | 2.433 | 0.011 |
| *Hist2h2aa2* | 2.433 | 0.011 |
| *Gm40749* | 3.111 | 0.039 |
| *Gm34702* | 4.376 | 0.039 |
| *LOC108167844* | 7.842 | 0.034 |

Genes assessed following criteria were selected as differentially expressed genes: Fold change ≥1.2, and *p*-value <0.05.
