# Supplementary material for: Colon Transcriptomics Reveals Sex-Dependent Metabolic Signatures in Response to 2-Amino-1-methyl-6-phenylimidazo[4,5-b]pyridine Treatment in C57BL/6N Mice
Source: Int J Mol Sci. 2020 Sep 10;21(18):6620. doi: 10.3390/ijms21186620 (PMC7555907; doi:10.3390/ijms21186620)
Supplement: Supplementary file 1 [file ijms-21-06620-s001.zip › Table S2.docx]

**Table S2.** A list of differentially expressed genes in colonic transcriptomics dataset of female mice.

| **Gene Symbol** | **Fold Change** | ***p*-Value** |
| --- | --- | --- |
| *Raet1e* | −4.608 | 0.044 |
| *Gm33309* | −3.884 | 0.020 |
| *Cyp2d12* | −2.780 | 0.038 |
| *Rdh9* | −2.662 | 0.017 |
| *Steap1* | −2.592 | 0.011 |
| *Slc46a1* | −2.456 | 0.018 |
| *LOC102640771* | −2.408 | 0.018 |
| *Cyp2c68* | −2.346 | 0.002 |
| *Eno3* | −2.317 | 0.006 |
| *Gm33224* | −2.235 | 0.050 |
| *Plk3* | −2.148 | 0.001 |
| *Zfp37* | −2.138 | 0.002 |
| *Kcnf1* | −2.126 | 0.035 |
| *Gm17197* | −2.103 | 0.022 |
| *Sh3d21* | −2.028 | 0.001 |
| *Pla2g10os* | −2.026 | 0.007 |
| *Mbnl3* | −2.020 | 0.028 |
| *Cyp2c69* | −2.008 | 0.039 |
| *Mptx1* | −2.003 | 0.007 |
| *Nrap* | −1.984 | 0.005 |
| *Ahnak2* | −1.982 | 0.043 |
| *Sycn* | −1.971 | 0.007 |
| *Slc16a3* | −1.968 | 0.020 |
| *Il18* | −1.921 | 0.008 |
| *Upb1* | −1.913 | 0.004 |
| *Trim46* | −1.909 | 0.014 |
| *Gm36193* | −1.873 | 0.049 |
| *S100a14* | −1.852 | 0.004 |
| *Xylt1* | −1.818 | 0.022 |
| *Slc35f2* | −1.806 | 0.005 |
| *Bmp2* | −1.791 | 0.001 |
| *Tmem218* | −1.788 | 0.010 |
| *Edn1* | −1.788 | 0.005 |
| *5430427M07Rik* | −1.785 | 0.027 |
| *Ggh* | −1.775 | 0.040 |
| *Hoxa11os* | −1.768 | 0.019 |
| *Gm36457* | −1.763 | 0.043 |
| *Ifnlr1* | −1.752 | 0.024 |
| *Gpr137b* | −1.743 | 0.003 |
| *Kyat1* | −1.727 | 0.000 |
| *Zfp775* | −1.726 | 0.023 |
| *Ces2g* | −1.725 | 0.002 |
| *Fnip2* | −1.715 | 0.012 |
| *Vill* | −1.701 | 0.022 |
| *Gna14* | −1.695 | 0.003 |
| *Samd8* | −1.693 | 0.005 |
| *Cela1* | −1.690 | 0.002 |
| *Csrnp1* | −1.690 | 0.000 |
| *Nr1i3* | −1.674 | 0.014 |
| *Rhbdl2* | −1.659 | 0.005 |
| *Wfdc2* | −1.656 | 0.003 |
| *Spats2l* | −1.634 | 0.002 |
| *Apobec1* | −1.625 | 0.026 |
| *C1qtnf6* | −1.615 | 0.008 |
| *Cyb5d2* | −1.611 | 0.038 |
| *Slc45a3* | −1.604 | 0.011 |
| *Ptrh1* | −1.604 | 0.036 |
| *Fam102b* | −1.597 | 0.001 |
| *Ap1s3* | −1.594 | 0.003 |
| *Tm6sf2* | −1.592 | 0.006 |
| *Mgat4a* | −1.573 | 0.017 |
| *Tnfrsf12a* | −1.571 | 0.049 |
| *Phlpp2* | −1.568 | 0.002 |
| *Gprc5a* | −1.565 | 0.014 |
| *Slc26a3* | −1.556 | 0.009 |
| *Chp2* | −1.554 | 0.009 |
| *Rhou* | −1.549 | 0.005 |
| *Igsf23* | −1.546 | 0.003 |
| *B3galt5* | −1.541 | 0.006 |
| *Pcgf5* | −1.539 | 0.000 |
| *3110001I22Rik* | −1.539 | 0.027 |
| *Hk2* | −1.529 | 0.013 |
| *Hif1a* | −1.525 | 0.000 |
| *Tirap* | −1.522 | 0.033 |
| *Clca4a* | −1.521 | 0.026 |
| *Batf2* | −1.519 | 0.015 |
| *Ppp1r3b* | −1.508 | 0.004 |
| *Tmem254a* | −1.503 | 0.047 |
| *Tmem254b* | −1.503 | 0.047 |
| *Tmem254c* | −1.503 | 0.047 |
| *Prdx6* | −1.501 | 0.006 |
| *Pgap1* | −1.501 | 0.048 |
| *Gm7710* | −1.500 | 0.010 |
| *Ccdc32* | −1.499 | 0.043 |
| *LOC108167895* | −1.498 | 0.030 |
| *Anxa3* | −1.494 | 0.003 |
| *Best2* | −1.492 | 0.034 |
| *Sytl1* | −1.484 | 0.001 |
| *Cpm* | −1.483 | 0.047 |
| *Tnfaip3* | −1.482 | 0.006 |
| *B4galnt3* | −1.474 | 0.040 |
| *Lyrm1* | −1.463 | 0.009 |
| *St3gal6* | −1.462 | 0.002 |
| *Lgalsl* | −1.457 | 0.008 |
| *Fads2* | −1.455 | 0.024 |
| *Pik3r3* | −1.454 | 0.018 |
| *Umad1* | −1.452 | 0.033 |
| *Tfrc* | −1.449 | 0.043 |
| *Trib1* | −1.447 | 0.025 |
| *Cklf* | −1.444 | 0.023 |
| *Upk1a* | −1.443 | 0.017 |
| *Hoxa7* | −1.436 | 0.003 |
| *Scnn1a* | −1.434 | 0.003 |
| *Tmem43* | −1.428 | 0.020 |
| *BC016579* | −1.427 | 0.033 |
| *Pmp22* | −1.421 | 0.005 |
| *Slc8b1* | −1.418 | 0.000 |
| *Serpinb1c* | −1.416 | 0.004 |
| *Stom* | −1.413 | 0.041 |
| *Hexb* | −1.411 | 0.025 |
| *Fam69b* | −1.409 | 0.014 |
| *Tmprss2* | −1.400 | 0.004 |
| *Rasef* | −1.400 | 0.004 |
| *Slc25a14* | −1.394 | 0.010 |
| *Itih5* | −1.392 | 0.007 |
| *Rin3* | −1.386 | 0.018 |
| *Nlrx1* | −1.386 | 0.014 |
| *Slc20a1* | −1.381 | 0.033 |
| *Rybp* | −1.367 | 0.010 |
| *Ndel1* | −1.366 | 0.008 |
| *Pdzk1ip1* | −1.363 | 0.034 |
| *Cxcl16* | −1.361 | 0.029 |
| *Epha2* | −1.361 | 0.003 |
| *Sun2* | −1.361 | 0.007 |
| *Tppp* | −1.356 | 0.003 |
| *Aadac* | −1.352 | 0.000 |
| *Svbp* | −1.351 | 0.017 |
| *4921531C22Rik* | −1.349 | 0.048 |
| *Ppa1* | −1.344 | 0.017 |
| *Efnb2* | −1.340 | 0.047 |
| *Srd5a1* | −1.330 | 0.023 |
| *Hrh1* | −1.330 | 0.049 |
| *BC030870* | −1.326 | 0.016 |
| *Fam160a1* | −1.325 | 0.036 |
| *Thnsl2* | −1.325 | 0.037 |
| *Fbxo32* | −1.322 | 0.015 |
| *Tmem53* | −1.322 | 0.024 |
| *Vkorc1l1* | −1.319 | 0.019 |
| *Rapgefl1* | −1.317 | 0.032 |
| *Rcor1* | −1.314 | 0.032 |
| *Mira* | −1.312 | 0.004 |
| *Soat1* | −1.312 | 0.027 |
| *Coq10b* | −1.312 | 0.028 |
| *Anxa1* | −1.311 | 0.029 |
| *Il22ra1* | −1.311 | 0.023 |
| *Mtf1* | −1.311 | 0.005 |
| *Tnfrsf11a* | −1.310 | 0.002 |
| *Spink1* | −1.310 | 0.044 |
| *Syk* | −1.310 | 0.009 |
| *Noct* | −1.309 | 0.010 |
| *Cep63* | −1.308 | 0.022 |
| *Tmem50b* | −1.307 | 0.029 |
| *Adh1* | −1.307 | 0.008 |
| *Hao2* | −1.307 | 0.019 |
| *Abat* | −1.301 | 0.032 |
| *Gm12942* | −1.300 | 0.018 |
| *9430038I01Rik* | −1.296 | 0.008 |
| *Snrnp25* | −1.293 | 0.028 |
| *Wwc2* | −1.293 | 0.017 |
| *Tmem79* | −1.291 | 0.042 |
| *Aste1* | −1.288 | 0.012 |
| *Slc16a1* | −1.287 | 0.034 |
| *Cdpf1* | −1.286 | 0.010 |
| *Litaf* | −1.285 | 0.006 |
| *Gpc4* | −1.284 | 0.018 |
| *Arhgap35* | −1.284 | 0.033 |
| *Ovol2* | −1.282 | 0.022 |
| *Mbd1* | −1.279 | 0.004 |
| *Gm15663* | −1.278 | 0.003 |
| *Insig1* | −1.273 | 0.002 |
| *Pfkfb4* | −1.272 | 0.004 |
| *Mpzl2* | −1.272 | 0.039 |
| *Lysmd1* | −1.271 | 0.041 |
| *Pdgfa* | −1.267 | 0.034 |
| *Nt5c3* | −1.264 | 0.027 |
| *Ccdc64b* | −1.262 | 0.036 |
| *Tspan5* | −1.257 | 0.007 |
| *Plp2* | −1.257 | 0.001 |
| *Rhob* | −1.257 | 0.016 |
| *Ptpn3* | −1.255 | 0.005 |
| *Cyp4f16* | −1.255 | 0.009 |
| *BC017643* | −1.254 | 0.008 |
| *Thop1* | −1.254 | 0.013 |
| *Synj2bp−cox16* | −1.253 | 0.044 |
| *Sowahc* | −1.251 | 0.025 |
| *Cers4* | −1.250 | 0.001 |
| *Mafk* | −1.244 | 0.030 |
| *Mrps6* | −1.242 | 0.003 |
| *Trpm6* | −1.242 | 0.049 |
| *Asb8* | −1.240 | 0.002 |
| *Gch1* | −1.239 | 0.037 |
| *S100a16* | −1.238 | 0.035 |
| *Tbc1d2* | −1.238 | 0.021 |
| *Utp14b* | −1.238 | 0.033 |
| *Cep112it* | −1.238 | 0.013 |
| *Ugcg* | −1.237 | 0.043 |
| *Phlpp1* | −1.236 | 0.015 |
| *Tmbim1* | −1.235 | 0.005 |
| *Mcl1* | −1.234 | 0.006 |
| *Cd320* | −1.234 | 0.050 |
| *Akap2* | −1.233 | 0.045 |
| *Ept1* | −1.232 | 0.004 |
| *Lgr4* | −1.229 | 0.020 |
| *Rep15* | −1.229 | 0.024 |
| *Tmem168* | −1.227 | 0.039 |
| *Pard6b* | −1.225 | 0.034 |
| *Sdsl* | −1.224 | 0.049 |
| *Sfxn3* | −1.224 | 0.008 |
| *Parm1* | −1.223 | 0.012 |
| *Rnd3* | −1.223 | 0.019 |
| *Kcnk5* | −1.222 | 0.006 |
| *Gskip* | −1.222 | 0.047 |
| *Met* | −1.222 | 0.040 |
| *Ripk4* | −1.221 | 0.012 |
| *Tm7sf2* | −1.221 | 0.025 |
| *Eef2k* | −1.220 | 0.003 |
| *Lrrc8a* | −1.220 | 0.002 |
| *Fam214b* | −1.220 | 0.002 |
| *Myo5c* | −1.219 | 0.044 |
| *LOC108167930* | −1.218 | 0.033 |
| *Specc1* | −1.217 | 0.016 |
| *Atp8a1* | −1.216 | 0.025 |
| *Ifrd1* | −1.214 | 0.016 |
| *Chchd4* | −1.214 | 0.033 |
| *Rwdd2b* | −1.214 | 0.002 |
| *Gmpr* | −1.213 | 0.028 |
| *Plekhh1* | −1.213 | 0.018 |
| *Tmtc2* | −1.212 | 0.002 |
| *Skil* | −1.212 | 0.023 |
| *Man1a2* | −1.210 | 0.029 |
| *Tnfrsf21* | −1.209 | 0.030 |
| *Fam8a1* | −1.209 | 0.037 |
| *LOC102633000* | −1.209 | 0.022 |
| *Rabif* | −1.208 | 0.007 |
| *Lmna* | −1.207 | 0.003 |
| *Txn1* | −1.205 | 0.025 |
| *Lamc2* | −1.204 | 0.008 |
| *Glul* | −1.204 | 0.006 |
| *Acap2* | −1.203 | 0.002 |
| *2410016O06Rik* | 1.201 | 0.018 |
| *Zfp36l2* | 1.202 | 0.001 |
| *Rnase4* | 1.203 | 0.004 |
| *Tspan17* | 1.203 | 0.015 |
| *Ttyh3* | 1.204 | 0.010 |
| *Cat* | 1.207 | 0.004 |
| *Golim4* | 1.207 | 0.002 |
| *Adh5* | 1.208 | 0.040 |
| *Prkag2* | 1.209 | 0.022 |
| *Gfod2* | 1.209 | 0.014 |
| *E430018J23Rik* | 1.210 | 0.022 |
| *Appl1* | 1.210 | 0.029 |
| *LOC108169130* | 1.212 | 0.036 |
| *Gm32949* | 1.213 | 0.007 |
| *Slc39a14* | 1.213 | 0.017 |
| *Hipk2* | 1.215 | 0.035 |
| *Zcchc11* | 1.216 | 0.049 |
| *Lig4* | 1.216 | 0.017 |
| *Scand1* | 1.216 | 0.044 |
| *Inpp1* | 1.217 | 0.028 |
| *Sdc2* | 1.217 | 0.049 |
| *Gm39469* | 1.220 | 0.030 |
| *Socs2* | 1.223 | 0.008 |
| *Zwint* | 1.226 | 0.013 |
| *Asns* | 1.226 | 0.039 |
| *Car1* | 1.227 | 0.040 |
| *Fzd8* | 1.230 | 0.005 |
| *Gm14391* | 1.236 | 0.049 |
| *Cyp4f40* | 1.237 | 0.011 |
| *Wfs1* | 1.238 | 0.008 |
| *Ppp2r3d* | 1.238 | 0.026 |
| *Sgpp1* | 1.238 | 0.025 |
| *Paqr5* | 1.239 | 0.042 |
| *2210404O09Rik* | 1.241 | 0.006 |
| *Elp2* | 1.242 | 0.004 |
| *Gm41949* | 1.242 | 0.041 |
| *Isoc1* | 1.243 | 0.030 |
| *Farp2* | 1.244 | 0.035 |
| *Oaf* | 1.248 | 0.024 |
| *Galk1* | 1.248 | 0.029 |
| *Ttyh2* | 1.251 | 0.001 |
| *Rbm48* | 1.252 | 0.044 |
| *Vat1* | 1.253 | 0.001 |
| *E330033B04Rik* | 1.254 | 0.037 |
| *Nr1h4* | 1.256 | 0.017 |
| *Fam35a* | 1.256 | 0.046 |
| *Spg20* | 1.258 | 0.030 |
| *Mast3* | 1.259 | 0.044 |
| *Proser2* | 1.262 | 0.026 |
| *Abhd6* | 1.262 | 0.042 |
| *Slc30a1* | 1.264 | 0.050 |
| *4933421O10Rik* | 1.266 | 0.005 |
| *Fam189b* | 1.267 | 0.049 |
| *Daglb* | 1.269 | 0.009 |
| *Lgmn* | 1.269 | 0.007 |
| *Gm15541* | 1.273 | 0.004 |
| *Lrrc8b* | 1.274 | 0.024 |
| *Wdr6* | 1.274 | 0.026 |
| *Ttll10* | 1.275 | 0.025 |
| *Peak1* | 1.276 | 0.030 |
| *LOC102640772* | 1.277 | 0.048 |
| *Rab3a* | 1.280 | 0.038 |
| *Gm44502* | 1.285 | 0.037 |
| *Pkig* | 1.289 | 0.023 |
| *Itpka* | 1.291 | 0.032 |
| *LOC108167878* | 1.293 | 0.043 |
| *2810408B13Rik* | 1.294 | 0.012 |
| *Idua* | 1.297 | 0.046 |
| *Chgb* | 1.298 | 0.008 |
| *Chga* | 1.299 | 0.009 |
| *Arrdc2* | 1.303 | 0.023 |
| *Ddhd2* | 1.309 | 0.043 |
| *Degs1* | 1.309 | 0.040 |
| *Gm36313* | 1.314 | 0.039 |
| *Parp11* | 1.316 | 0.008 |
| *Hpse* | 1.322 | 0.045 |
| *Fcho1* | 1.323 | 0.016 |
| *Naaladl1* | 1.326 | 0.018 |
| *Cpne8* | 1.326 | 0.002 |
| *Col18a1* | 1.329 | 0.019 |
| *Jrkl* | 1.329 | 0.048 |
| *Trim14* | 1.334 | 0.011 |
| *Ddx58* | 1.335 | 0.018 |
| *Nin* | 1.336 | 0.034 |
| *4930526I15Rik* | 1.338 | 0.028 |
| *Cyp4v3* | 1.340 | 0.039 |
| *Acsf2* | 1.343 | 0.022 |
| *Gpt* | 1.348 | 0.011 |
| *Smco4* | 1.349 | 0.044 |
| *Ang* | 1.355 | 0.005 |
| *Rarg* | 1.366 | 0.018 |
| *Samd10* | 1.367 | 0.009 |
| *Tmem184c* | 1.367 | 0.027 |
| *Acaa1b* | 1.373 | 0.013 |
| *Prex1* | 1.373 | 0.049 |
| *Tbc1d4* | 1.375 | 0.010 |
| *Guca2b* | 1.377 | 0.034 |
| *Cfd* | 1.381 | 0.026 |
| *Gm41284* | 1.384 | 0.005 |
| *LOC108168022* | 1.384 | 0.042 |
| *Cnnm2* | 1.387 | 0.045 |
| *Tph1* | 1.390 | 0.022 |
| *Cox18* | 1.395 | 0.007 |
| *1700008J07Rik* | 1.400 | 0.002 |
| *4932416H05Rik* | 1.401 | 0.032 |
| *Fbln2* | 1.403 | 0.036 |
| *Gm34489* | 1.404 | 0.012 |
| *Gm13157* | 1.404 | 0.033 |
| *Tmem25* | 1.408 | 0.005 |
| *Agt* | 1.410 | 0.035 |
| *Pla2g6* | 1.417 | 0.018 |
| *Slco2b1* | 1.417 | 0.014 |
| *Sccpdh* | 1.418 | 0.038 |
| *Pcsk1n* | 1.424 | 0.013 |
| *Fgfrl1* | 1.427 | 0.001 |
| *Sulf2* | 1.431 | 0.042 |
| *Gm40371* | 1.431 | 0.015 |
| *Lipe* | 1.435 | 0.028 |
| *Nid1* | 1.442 | 0.045 |
| *Ace2* | 1.442 | 0.046 |
| *Camk2b* | 1.443 | 0.002 |
| *Pbld1* | 1.453 | 0.029 |
| *Fads1* | 1.454 | 0.004 |
| *Tnfaip8l1* | 1.455 | 0.030 |
| *Sap30* | 1.455 | 0.016 |
| *Gpd1* | 1.469 | 0.002 |
| *Atp1a2* | 1.470 | 0.043 |
| *Zfp703* | 1.470 | 0.030 |
| *Ces1f* | 1.477 | 0.047 |
| *Fbxw17* | 1.478 | 0.005 |
| *Myef2* | 1.480 | 0.017 |
| *Insl6* | 1.482 | 0.040 |
| *1110046J04Rik* | 1.485 | 0.034 |
| *Atxn7l1* | 1.486 | 0.010 |
| *9430083A17Rik* | 1.489 | 0.031 |
| *Gm17066* | 1.492 | 0.016 |
| *2310016G11Rik* | 1.498 | 0.041 |
| *Fabp4* | 1.499 | 0.031 |
| *Rac2* | 1.507 | 0.045 |
| *Slc51b* | 1.508 | 0.045 |
| *Fmo5* | 1.534 | 0.029 |
| *Sesn1* | 1.537 | 0.009 |
| *Gm10814* | 1.537 | 0.040 |
| *Acvr1c* | 1.540 | 0.011 |
| *Sst* | 1.541 | 0.018 |
| *Tctex1d2* | 1.543 | 0.030 |
| *Abcc9* | 1.544 | 0.011 |
| *Ifit3* | 1.547 | 0.016 |
| *Gm13248* | 1.548 | 0.033 |
| *Cebpa* | 1.552 | 0.003 |
| *Ugt1a6a* | 1.553 | 0.044 |
| *Slc2a9* | 1.554 | 0.049 |
| *Nceh1* | 1.560 | 0.007 |
| *Car3* | 1.563 | 0.036 |
| *Lrfn3* | 1.563 | 0.009 |
| *Flrt3* | 1.567 | 0.048 |
| *Fyb* | 1.570 | 0.012 |
| *4632404H12Rik* | 1.572 | 0.036 |
| *Eepd1* | 1.572 | 0.047 |
| *Jag2* | 1.576 | 0.028 |
| *Cib2* | 1.576 | 0.042 |
| *9630028B13Rik* | 1.580 | 0.046 |
| *1700030C10Rik* | 1.582 | 0.004 |
| *Ggt1* | 1.588 | 0.016 |
| *Acot4* | 1.589 | 0.013 |
| *Fgl2* | 1.596 | 0.025 |
| *Dsel* | 1.601 | 0.004 |
| *Bche* | 1.601 | 0.021 |
| *LOC108167320* | 1.613 | 0.041 |
| *Gm44505* | 1.621 | 0.002 |
| *Ppp3cc* | 1.628 | 0.003 |
| *Anks6* | 1.633 | 0.015 |
| *Tgfbi* | 1.634 | 0.015 |
| *Slc43a2* | 1.635 | 0.017 |
| *Stra6l* | 1.636 | 0.017 |
| *Prodh* | 1.639 | 0.004 |
| *Cyp2d26* | 1.639 | 0.012 |
| *Slc25a37* | 1.653 | 0.046 |
| *Limd2* | 1.658 | 0.040 |
| *Asap3* | 1.663 | 0.042 |
| *Sema7a* | 1.670 | 0.027 |
| *Tubb2b* | 1.677 | 0.032 |
| *Gdpd2* | 1.683 | 0.044 |
| *Cmpk2* | 1.690 | 0.011 |
| *Aspg* | 1.697 | 0.014 |
| *Cd36* | 1.703 | 0.026 |
| *Gm4791* | 1.704 | 0.043 |
| *Adipoq* | 1.725 | 0.034 |
| *Tspan6* | 1.755 | 0.028 |
| *Mboat2* | 1.756 | 0.038 |
| *Lpl* | 1.758 | 0.013 |
| *Gm40210* | 1.761 | 0.025 |
| *LOC108168395* | 1.766 | 0.018 |
| *Nfil3* | 1.780 | 0.006 |
| *Tlr2* | 1.810 | 0.046 |
| *Lpcat4* | 1.816 | 0.024 |
| *Ccl25* | 1.820 | 0.003 |
| *Itga2* | 1.824 | 0.038 |
| *Ppara* | 1.833 | 0.022 |
| *Phospho1* | 1.833 | 0.019 |
| *Ttyh1* | 1.841 | 0.042 |
| *Acot2* | 1.878 | 0.040 |
| *Xpnpep2* | 1.889 | 0.005 |
| *Ces1g* | 1.905 | 0.004 |
| *Slc18a1* | 1.920 | 0.006 |
| *Cd44* | 1.922 | 0.024 |
| *C1qtnf3* | 1.925 | 0.024 |
| *4930555F03Rik* | 1.930 | 0.002 |
| *Il18bp* | 1.953 | 0.040 |
| *Gm2061* | 1.960 | 0.011 |
| *Gm38524* | 2.003 | 0.049 |
| *Tmem65* | 2.034 | 0.040 |
| *Cd7* | 2.039 | 0.018 |
| *Slc38a9* | 2.083 | 0.049 |
| *Nes* | 2.098 | 0.030 |
| *Ifi47* | 2.102 | 0.032 |
| *Ccl5* | 2.109 | 0.016 |
| *Nt5dc2* | 2.110 | 0.034 |
| *Mfsd4b3* | 2.121 | 0.008 |
| *Mfap3l* | 2.150 | 0.023 |
| *Slc16a9* | 2.151 | 0.002 |
| *Gm40448* | 2.192 | 0.036 |
| *Fah* | 2.195 | 0.010 |
| *G0s2* | 2.207 | 0.024 |
| *AA414768* | 2.212 | 0.009 |
| *Gm42245* | 2.239 | 0.025 |
| *Arg2* | 2.248 | 0.001 |
| *Mypop* | 2.292 | 0.011 |
| *Trbc2* | 2.314 | 0.041 |
| *Bend7* | 2.340 | 0.017 |
| *Slc10a2* | 2.344 | 0.011 |
| *Thbs2* | 2.361 | 0.019 |
| *Angptl4* | 2.411 | 0.007 |
| *Cd3g* | 2.439 | 0.005 |
| *Ddah1* | 2.462 | 0.036 |
| *Gm32474* | 2.516 | 0.010 |
| *Gm40572* | 2.521 | 0.019 |
| *Enpep* | 2.601 | 0.015 |
| *Fam3c* | 2.662 | 0.034 |
| *Nkain1* | 2.741 | 0.001 |
| *Ido1* | 2.804 | 0.031 |
| *Fgd1* | 2.819 | 0.024 |
| *9330104G04Rik* | 2.933 | 0.028 |
| *D430020J02Rik* | 2.935 | 0.007 |
| *Adhfe1* | 3.217 | 0.020 |
| *Rab37* | 3.387 | 0.048 |
| *Gm11816* | 3.481 | 0.028 |
| *Tnfsf10* | 3.499 | 0.020 |
| *Prlr* | 3.577 | 0.014 |
| *1700020L24Rik* | 3.941 | 0.005 |
| *Gm32728* | 4.084 | 0.000 |
| *Gm11437* | 5.085 | 0.046 |
| *Gm13657* | 5.320 | 0.003 |
| *Tlr5* | 6.220 | 0.019 |

Genes assessed following criteria were selected as differentially expressed genes: Fold change ≥1.2, and *p*−value <0.05.
