# Supplementary material for: Colon Transcriptomics Reveals Sex-Dependent Metabolic Signatures in Response to 2-Amino-1-methyl-6-phenylimidazo[4,5-b]pyridine Treatment in C57BL/6N Mice
Source: Int J Mol Sci. 2020 Sep 10;21(18):6620. doi: 10.3390/ijms21186620 (PMC7555907; doi:10.3390/ijms21186620)
Supplement: Supplementary file 1 [file ijms-21-06620-s001.zip › Table S3-S8.docx]

This document contains: **Tables S3–S8**

**Table S3.** Significantly overrepresented Protein Annotation through Evolutionary Relationships (PANTHER) gene ontology (GO) for colonic transcriptome of 2-amino-1-methyl-6-phenylimidazo[4,5-b]pyridine (PhIP)-treated male and female mice ^1^.

| **Category** | **PANTHER GO Terms** | **Number of Genes in Reference Genome Dataset** | **Number of Genes in Dataset** | **Fold Enrichment** | **FDR** |
| --- | --- | --- | --- | --- | --- |
| Biological Process | Exogenous drug catabolic process (GO:0042738) | 53 | 7 | 7.31 | 0.0462 |
|  | Long-chain fatty acid metabolic process (GO:0001676) | 95 | 11 | 6.41 | 0.00312 |
|  | Unsaturated fatty acid metabolic process (GO:0033559) | 97 | 10 | 5.7 | 0.0134 |
|  | Xenobiotic metabolic process (GO:0006805) | 107 | 10 | 5.17 | 0.0269 |
|  | Icosanoid metabolic process (GO:0006690) | 107 | 10 | 5.17 | 0.0259 |
|  | Fatty acid metabolic process (GO:0006631) | 309 | 21 | 3.76 | 0.00095 |
|  | Monocarboxylic acid metabolic process (GO:0032787) | 467 | 27 | 3.2 | 0.00055 |
|  | Lipid biosynthetic process (GO:0008610) | 416 | 23 | 3.06 | 0.00346 |
|  | Cellular lipid metabolic process (GO:0044255) | 801 | 44 | 3.04 | 1.52E−06 |
|  | Lipid metabolic process (GO:0006629) | 1062 | 56 | 2.92 | 3.63E−08 |
| Molecular Function | Carboxylic ester hydrolase activity (GO:0052689) | 159 | 12 | 4.18 | 0.02440 |
|  | Nucleoside-triphosphatase regulator activity (GO:0060589) | 295 | 17 | 3.19 | 0.02210 |
|  | Enzyme activator activity (GO:0008047) | 454 | 21 | 2.56 | 0.04370 |
|  | Hydrolase activity, acting on ester bonds (GO:0016788) | 737 | 34 | 2.55 | 9.47E−05 |
|  | Metal ion binding (GO:0046872) | 3499 | 94 | 1.49 | 0.02840 |
|  | Catalytic activity (GO:0003824) | 5693 | 152 | 1.48 | 9.47E−05 |
|  | Cation binding (GO:0043169) | 3598 | 96 | 1.48 | 0.02860 |
|  | Ion binding (GO:0043167) | 5483 | 143 | 1.44 | 0.00104 |
|  | Molecular_function (GO:0003674) | 20214 | 389 | 1.06 | 0.00660 |
|  | Signaling receptor activity (GO:0038023) | 2334 | 13 | 0.31 | 0.00011 |
| Cellular Component | Mitochondrion (GO:0005739) | 1802 | 58 | 1.78 | 0.0075 |
|  | Intracellular membrane-bounded organelle (GO:0043231) | 10278 | 250 | 1.35 | 7.31E−08 |
|  | Membrane-bounded organelle (GO:0043227) | 11027 | 268 | 1.34 | 5.29E−09 |
|  | Cytoplasm (GO:0005737) | 10961 | 265 | 1.34 | 1.26E−08 |
|  | Intracellular organelle (GO:0043229) | 11952 | 288 | 1.33 | 3.10E−10 |
|  | Organelle (GO:0043226) | 12299 | 294 | 1.32 | 3.96E−10 |
|  | Intracellular (GO:0005622) | 13692 | 325 | 1.31 | 3.71E−13 |
|  | Cellular anatomical entity (GO:0110165) | 18643 | 371 | 1.1 | 0.00031 |

^1^ Short-listed differentially expressed transcripts were subjected to the PANTHER analysis for over-represented changes with transitions between control and PhIP-treated group. Displayed data in the descending order of fold enrichment.

**Table S4.** Significantly overrepresented Protein Annotation through Evolutionary Relationships (PANTHER) gene ontology (GO) terms for colonic transcriptome of 2-amino-1-methyl-6-phenylimidazo[4,5-b]pyridine (PhIP)-treated male mice ^1^.

| **Category** | **PANTHER GO Terms** | **Number of Genes in Reference Genome Dataset** | **Number of Genes in Dataset** | **Fold Enrichment** | **False Discovery Rate** |
| --- | --- | --- | --- | --- | --- |
| Biological Process | Electron transport chain (GO:0022900) | 30 | 6 | 8.71 | 0.01340 |
|  | Respiratory electron transport chain (GO:0022904) | 30 | 6 | 8.71 | 0.01410 |
|  | Energy coupled proton transport, down electrochemical gradient (GO:0015985) | 36 | 7 | 8.47 | 0.00907 |
|  | ATP synthesis coupled proton transport (GO:0015986) | 36 | 7 | 8.47 | 0.01010 |
|  | ATP biosynthetic process (GO:0006754) | 36 | 7 | 8.47 | 0.01130 |
|  | Proton transmembrane transport (GO:1902600) | 47 | 9 | 8.34 | 0.00411 |
|  | Oxidative phosphorylation (GO:0006119) | 27 | 5 | 8.07 | 0.04680 |
|  | Purine ribonucleoside triphosphate metabolic process (GO:0009205) | 38 | 7 | 8.03 | 0.00946 |
|  | Purine ribonucleoside triphosphate biosynthetic process (GO:0009206) | 38 | 7 | 8.03 | 0.01020 |
|  | Purine nucleoside triphosphate biosynthetic process (GO:0009145) | 38 | 7 | 8.03 | 0.01120 |
| Molecular Function | Cytochrome-c oxidase activity (GO:0004129) | 12 | 4 | 14.52 | 0.0244 |
|  | Structural constituent of ribosome (GO:0003735) | 128 | 20 | 6.81 | 3.67E-08 |
|  | Structural molecule activity (GO:0005198) | 218 | 21 | 4.2 | 1.57E−05 |
|  | Signaling receptor binding (GO:0005102) | 734 | 4 | 0.24 | 0.0264 |
|  | Molecular transducer activity (GO:0060089) | 1349 | 7 | 0.23 | 3.72E−05 |
|  | Signaling receptor activity (GO:0038023) | 1282 | 3 | 0.1 | 2.20E−07 |
|  | Transmembrane signaling receptor activity (GO:0004888) | 1182 | 1 | 0.04 | 3.00E−08 |
|  | G protein-coupled receptor activity (GO:0004930) | 405 | 0 | <0.01 | 0.0205 |
| Cellular Component | Respiratory chain complex I (GO:0045271) | 27 | 8 | 12.91 | 0.000031 |
|  | Mitochondrial respiratory chain complex I (GO:0005747) | 24 | 7 | 12.71 | 0.000107 |
|  | NADH dehydrogenase complex (GO:0030964) | 28 | 8 | 12.45 | 0.000034 |
|  | Proton-transporting ATP synthase complex (GO:0045259) | 11 | 3 | 11.88 | 0.033300 |
|  | Mitochondrial large ribosomal subunit (GO:0005762) | 17 | 4 | 10.25 | 0.012500 |
|  | Organellar ribosome (GO:0000313) | 30 | 6 | 8.71 | 0.002020 |
|  | Mitochondrial ribosome (GO:0005761) | 30 | 6 | 8.71 | 0.001970 |
|  | Mitochondrial respirasome (GO:0005746) | 41 | 8 | 8.5 | 0.000244 |
|  | Respirasome (GO:0070469) | 48 | 9 | 8.17 | 0.000105 |
|  | Oxidoreductase complex (GO:1990204) | 43 | 8 | 8.11 | 0.000319 |

^1^ Short-listed differentially expressed transcripts were subjected to the PANTHER analysis for over-represented changes with transitions between control and PhIP-treated group. Displayed data in the descending order of fold enrichment.

Short-listed differentially expressed transcripts were subjected to the PANTHER analysis for over-represented changes with transitions between control and PhIP-treated mice.

**Table S5.** Significantly overrepresented PANTHER gene ontology (GO) terms for colonic transcriptome of PhIP-treated female mice ^1^.

| **Category** | **PANTHER GO Terms** | **Number of Genes in Reference Genome Dataset** | **Number of Genes in Dataset** | **Fold Enrichment** | **FDR** |
| --- | --- | --- | --- | --- | --- |
| Biological Process | Negative regulation of establishment of protein localization (GO:1904950) | 3 | 3 | 51.42000 | 0.01820 |
|  | Negative regulation of protein transport (GO:0051224) | 3 | 3 | 51.42000 | 0.01700 |
|  | Monocarboxylic acid transport (GO:0015718) | 36 | 6 | 8.57000 | 0.01660 |
|  | Carboxylic acid transport (GO:0046942) | 95 | 9 | 4.87000 | 0.01960 |
|  | Organic acid transport (GO:0015849) | 96 | 9 | 4.82000 | 0.02000 |
|  | Small molecule catabolic process (GO:0044282) | 108 | 10 | 4.76000 | 0.01320 |
|  | Monocarboxylic acid metabolic process (GO:0032787) | 181 | 16 | 4.55000 | 0.00045 |
|  | Cellular amino acid metabolic process (GO:0006520) | 123 | 10 | 4.18000 | 0.02490 |
|  | Lipid metabolic process (GO:0006629) | 401 | 31 | 3.98000 | 6.54E−07 |
|  | Carboxylic acid metabolic process (GO:0019752) | 331 | 24 | 3.73000 | 0.0001 |
| Molecular Function | Lipase activity (GO:0016298) | 82 | 8 | 5.02 | 0.04320 |
|  | Anion transmembrane transporter activity (GO:0008509) | 221 | 14 | 3.26 | 0.03220 |
|  | Hydrolase activity (GO:0016787) | 1631 | 59 | 1.86 | 0.00185 |
|  | Catalytic activity (GO:0003824) | 3715 | 115 | 1.59 | 0.00019 |

^1^ Short-listed differentially expressed transcripts were subjected to the PANTHER analysis for over-represented changes with transitions between control and PhIP-treated group. Displayed data in the descending order of fold enrichment.

Short-listed differentially expressed transcripts were subjected to the PANTHER analysis for over-represented changes with transitions between control and PhIP-treated mice.

**Table S6.** RNA Integrity Number (RIN) for colonic RNA samples used for transcriptomics.

| **Animal** | **RIN** |
| --- | --- |
| M-CON 1 | 7.7 |
| M-CON 2 | 7.3 |
| M-CON 3 | 8.5 |
| M-PhIP 1 | 7.9 |
| M-PhIP 2 | 7.8 |
| M-PhIP 3 | 8.1 |
| F-CON 1 | 7.8 |
| F-CON 2 | 8.1 |
| F-CON 3 | 7.6 |
| F-PhIP 1 | 7.1 |
| F-PhIP 2 | 7.9 |
| F-PhIP 3 | 7.7 |

**Table S7.** A list of differentially expressed genes in common between male and female colonic transcriptomics dataset.

| **Gene Symbol** | **Fold Change (Female)** | **Fold Change (Male)** |
| --- | --- | --- |
| *Acbd3* | −−1.07 | −1.16 |
| *Anp32a* | 1.11 | 1.09 |
| *C1qtnf6* | −1.62 | −1.97 |
| *Car1* | 1.23 | 1.42 |
| *Cc2d1a* | −1.11 | −1.07 |
| *Ces1d* | 1.16 | 1.21 |
| *Cpm* | −1.48 | −1.21 |
| *Dennd6a* | −1.10 | −1.23 |
| *H2afj* | 1.15 | 1.40 |
| *Kcnf1* | −2.13 | −1.94 |
| *Kpna4* | −1.08 | −1.07 |
| *Lamc2* | −1.20 | −1.27 |
| *Lig4* | 1.22 | −1.20 |
| *Mbnl3* | −2.02 | −2.68 |
| *Mrps34* | 1.10 | 1.24 |
| *Ppargc1a* | −1.11 | −1.26 |
| *Ppp1r3b* | −1.51 | −2.19 |
| *Preb* | 1.06 | 1.08 |
| *Rab27b* | −1.12 | −1.45 |
| *Rabep1* | 1.17 | −1.18 |
| *Rapgefl1* | −1.32 | −1.33 |
| *Rrnad1* | 1.05 | 1.11 |
| *Scamp1* | −1.17 | −1.12 |
| *Scand1* | 1.22 | 1.35 |
| *Sgpp2* | −1.15 | −1.12 |
| *Slc46a1* | −2.46 | −1.63 |
| *Tctex1d2* | 1.54 | 1.85 |
| *Tmem168* | −1.23 | −1.25 |
| *Usp28* | −1.20 | −1.25 |

**Table S8.** Primer information.

| **Primer** | **Manufacturer** | **Assay ID** | **RefSeqNumber** |
| --- | --- | --- | --- |
| *Gapdh* | IDT | Mm.PT.39a.1 | NM_008084 |
| *Cox7a1* |  | Mm.PT.58.17219080 | NM_009944 |
| *Ndufa5* |  | Mm.PT.58.8505610 | NM_026614 |
| *Fis1* |  | Mm.PT.56a.21878911 | NM_025562 |
| *Ago4* |  | Mm.PT.58.10496728 | NM_153177 |
| *Timm13* |  | Mm.PT.58.7453172.g | NM_013895 |
| *Lpl* |  | Mm.PT.58.46006099 | NM_008509 |
| *Lipe* |  | Mm.PT.58.6342082 | NM_010719 |
| *Cebpa* |  | Mm.PT.58.30061639.g | NM_007678 |
| *Fabp4* |  | Mm.PT.58.43866459 | NM_024406 |
| *Hif1a* |  | Mm.PT.58.11211292 | NM_010431 |
| *Bmp2* |  | Mm.PT.58.10419414 | NM_007553 |
| *Atp5po* | Life Technologies | Mm05832152-g1 | NM_138597.2 |

Short-listed differentially expressed transcripts were subjected to the PANTHER analysis for over-represented changes with transitions between control and PhIP-treated mice.
